# Prevalence of *Batrachochytrium dendrobatidis* in Amphibians From 2000 to 2021: A Global Systematic Review and Meta-Analysis

**DOI:** 10.3389/fvets.2021.791237

**Published:** 2021-12-17

**Authors:** Zhongle Li, Qi Wang, Keping Sun, Jiang Feng

**Affiliations:** ^1^Jilin Provincial Key Laboratory of Animal Resource Conservation and Utilization, Northeast Normal University, Changchun, China; ^2^College of Animal Science and Technology, Jilin Agricultural University, Changchun, China; ^3^Key Laboratory of Vegetation Ecology, Ministry of Education, Changchun, China

**Keywords:** *Batrachochytrium dendrobatidis*, meta-analysis, amphibians, chytridiomycosis, prevalence

## Abstract

Chytridiomycosis is an amphibian fungal disease caused by *Batrachochytrium dendrobatidis* (*Bd*), which has caused large-scale death and population declines on several continents around the world. To determine the current status of *Bd* infection in amphibians, we conducted a global meta-analysis. Using PubMed, ScienceDirect, SpringerLink, China National Knowledge Infrastructure (CNKI) and Wanfang database searches, we retrieved a total of 111 articles from 2000 to 2021. Based on these, we estimated the *Bd* prevalence to be 18.54% (95% CI: 13.76–20.52) in current extent amphibians. Among these populations, the prevalence of *Bd* in Asia was the lowest at 7.88% (95% CI: 1.92–8.71). Further, no *Bd* infection was found in Vietnam. However, the prevalence of *Bd* in Oceania was the highest at 36.34% (95% CI: 11.31–46.52). The *Bd* prevalence in Venezuela was as high as 49.77% (95% CI: 45.92–53.62). After 2009, the global *Bd* prevalence decreased to 18.91% (95% CI: 13.23–21.56). The prevalence of *Bd* in epizootic populations was significantly higher than enzootic populations. The highest prevalence of *Bd* was detected with real-time PCR at 20.11% (95% CI: 13.12–21.38). The prevalence of *Bd* in frogs was the highest at 20.04% (95% CI: 13.52–21.71), and this different host was statistically significant (*P* < 0.05). At the same time, we analyzed the geographic factors (longitude, latitude, elevation, rainfall and temperature) that impacted the fungal prevalence in amphibians. Our meta-analysis revealed that factors including region, disease dynamic, detection method, host and climate may be sources of the observed heterogeneity. These results indicate that chytridiomycosis was a consistent threat to amphibians from 2000 to 2021. Based on different habitat types and geographical conditions, we recommend formulating corresponding control plans and adopting reasonable and efficient biological or chemical methods to reduce the severity of such diseases.

## Highlights

- The highest prevalence of *Bd* was in Oceania, followed by South America.
- The prevalence of amphibian chytridiomycosis has decreased over the past decade.
- Climate and geographical conditions may be the main risk factors for amphibian chytridiomycosis.

## Introduction

Over the last four decades, ~43% of amphibian species have become threatened with extinction worldwide (1, 2). Habitat destruction (3), natural environment pollution (4), climate change (5) and emerging infectious diseases (6) are all potential causes of morbidity and mortality. Chytridiomycosis is the disease caused by *Batrachochytrium dendrobatidis* (*Bd*) and has become a major menace to amphibians, leading to the decline and extinction of amphibians worldwide (7, 8).

*Bd* invades epidermal cells and grows the outer keratinized layers, causing skin thickening, erosions and ulcerations, disrupting the transport of water, oxygen and ions, and eventually leading to death (9). This pathogen has been detected in ~700 amphibian species (10) and occurs in different regions around the world, including Europe, Oceania, Africa, North America and South America (11, 12), particularly in the Neotropics and Australia (13, 14). *Bd* may be one of the most seriously infectious diseases currently threatening biodiversity (8) and is now listed as an important epidemic disease by the World Organization for Animal Health (15). Currently, there are several methods to control the spread of *Bd*, such as use of fungicides, bioaugmentation (microorganisms that inhibit the growth of *Bd*) and vaccination. The first two methods may be considered risky for other species due to their broad spectrum, and the effectiveness and safety of vaccination still need be further validation for controlling chytridiomycosis in the field (16, 17).

*Bd* is regarded as a generalist pathogen (18) and is distributed across different ecosystems (19, 20). The prevalence and infection intensity of *Bd* are associated with abiotic factors (21) and may vary according to host species, age and individual susceptibility (22). Currently, there are several *Bd* lineages, mainly divided into enzootic and epizootic lineages, where the enzootic lineages has a lower virulence probably due to its smaller zoosporangium size, and more limited geographic distributions vs. epizootic lineages (23–25). Meanwhile, the inherent characteristics of amphibians (behavior and immunity) and environmental features (vegetation coverage, temperature, rainfall, seasonality, and geographical conditions) can predict dynamic changes of diseases in natural populations (26, 27).

Amphibians are a critical links in ecosystem food chains, as they are able to prey on pests, effectively protect crops and play an important role in maintaining the ecosystem balance (18, 28). In addition, some amphibians are edible while others have high medicinal and scientific research value (29, 30). Considering the serious impact of chytridiomycosis on the health of amphibians, there are still limited articles on the overall assessment of the factors that potentially influence the prevalence of *Bd*. Thus, we conducted a global systematic review and meta-analysis on *Bd* infection over the last 21 years. The sampling year, geographic location, disease dynamic, detection method, season, host, age, International Union for the Conservation of Nature (IUCN) classification, sampling source and other potential risk factors along with several geographic factors were collected and analyzed to determine which were related to *Bd* prevalence in amphibians. This study could support ongoing conservation efforts for amphibian chytridiomycosis.

## Materials and Methods

### Article Search Strategy

We used the preferred reporting items for systematic reviews and meta-analysis (PRISMA) algorithm to select reasonable study reports (31). In this study, we searched for articles with the prevalence of *Bd* in amphibians worldwide available from five literature databases, including PubMed, ScienceDirect, SpringerLink, China National Knowledge Infrastructure (CNKI) and Wanfang Databases (the retrieval time was from Jan 1, 2000, to August 18, 2021). In PubMed, we used the MeSH terms “*Batrachochytrium dendrobatidis*,” “Prevalence,” and “Amphibians” for our searches. Then we queried the corresponding MeSH term “*Batrachochytrium dendrobatidis*” using the free words “Chytridiomycosis” and “Chytrid.” Simultaneously, the Boolean operator “AND” to connect MeSH terms and “OR” to connect the free words were used. Finally, the search formula “(*Batrachochytrium dendrobatidis* OR Chytridiomycosis OR Chytrid) AND (Amphibians) AND (Prevalence)” was used. In the ScienceDirect and SpringerLink databases, we used the same search formula. Meanwhile, we used the keywords “(*Batrachochytrium dendrobatidis*) AND (Amphibians)” for advanced searches and added a fuzzy search and synonym expansion in two Chinese databases. Moreover, we did not include unpublished data and did not seek additional information from the authors of published articles. Endnote X9 (version 9.3.1) was used to sort out the retrieved citations.

### Selection and Exclusion Criteria

We adopted the following inclusion criteria: (1) the study was aimed to estimate the prevalence of *Bd* from amphibians inhabiting the natural environment; (2) the study presented the number of examined species and the number of *Bd* positive species to evaluate the prevalence (the number of individuals positive to *Bd*/total number of individuals examined); (3) the sample size of the study was more than 60 (32); (4) the study design was cross-sectional for estimating the prevalence in disease studies. Moreover, the following exclusion criteria were used: (1) the study had internal data conflicts (the data described in the conclusion not corresponding to the actual number of tests); (2) the study included museum samples or specimen (unclear source of samples); (3) the study used experimental group data; (4) the study was conducted earlier than 2000; or (5) the study presented dead samples.

### Data Extraction and Analysis

Based on the acquired studies, we extracted the information using standardized data collection forms: first author, publication year, sampling date, continent of the study, country of the study, disease dynamic (the process of the spread of diseases from epizootic phase to enzootic phase), detection method, host classification (order), geographical factors, collection season, age, International Union for Conservation of Nature (IUCN) category, sample source, total number of amphibian samples and the number of *Bd* positive samples. The geographic factor data were based on weather stations collected from the NOAA National Centers for Environmental Information (https://gis.ncdc.noaa.gov/maps/ncei/cdo/monthly), including longitude, latitude, elevation, monthly average rainfall and monthly average temperature.

### Quality Assessment

The quality of the publications was scored with reference to a previously published method from the Grading of Recommendations Assessment, Development and Evaluation method (GRADE) (33, 34). The scoring criteria had the following conditions: clear detection method, definite random sampling, definite sampling time, clear sampling method and four or more risk factors evaluated in collection articles. Each condition was scored with one point. High-quality articles were valued to have 4–5 points, and medium-quality articles had 2–3 points. Low-quality articles had only 1 or 0 points (34). The scoring of an article did not represent the level of its research content, but was used to assess risk bias.

### Statistical Analysis

All statistical computing was conducted in R version 4.0.2 (35). Based on previous studies, we chose arcsine transformation (PFT) data to be closer to the normal distribution before our meta-analysis (*W* = 0.96961; *P* < 0.05; Supplementary Table 1) (36).

The formulas for PFT were as follows: *t* = arcsin {sqrt [r/(n + 1)]} + arcsin {sqrt [(r + 1)/(n + 1)]}, note: *t* = transformed prevalence; *n* = sample size; *r* = positive number; se = standard error (37). We calculated the heterogeneity between studies using Cochran's Q, *I*^2^ statistics and the χ^2^ test, where I^2^>50% indicates a significant degree of heterogeneity. Based on the obvious heterogeneity from the selected articles, we chose a random model for meta-analysis (38). In order to examine the potential sources of heterogeneity, we analyzed the research data using subgroup analyses and univariate regression analysis to identify potential risk factors predictive of heterogeneity. Forest plots were used to visually evaluate the overall results of metaanalysis. The studies of publication bias were explained with funnel plots and an Egger's test. Finally, we used sensitivity analysis to estimate the stability of our results (39).

We further studied the potential sources of heterogeneity from each subgroup (40), including the geographic region (Africa, Asia, Europe, North America, Oceania and South America), the sampling year (2000–2008 and after 2009), disease dynamic (enzootic and epizootic), detection method (conventional PCR, nested PCR, real-time PCR, histopathology examination and LAMP), host (caecilian, frog, salamander, toad, newt), season (spring, summer, autumn and winter), age (adult, subadult, juvenile and tadpole), IUCN category (least concern, near threatened, vulnerable, endangered, and critically endangered), sample source (forest, park, lake, pond, stream, and other sources), and quality level (middle quality and high quality).

We also analyzed the subgroup of geographical risk factors that influenced the heterogeneity (41), based on latitude (north latitude 0–15°, north latitude 15–30°, north latitude 30–60°, south latitude 0–20°, and south latitude 20–40°), longitude (east longitude 0–80°, east longitude 80–120°, east longitude 120–160°, west longitude 0–100°, and west longitude 100–160°), elevation (0–100 m, 100–500 m, 500–1700 m, and 1700–5000 m), monthly average rainfall (0–50 mm, 50–100 mm, 100–150 mm, and >150 mm) and monthly average temperature (5–16°C, 17–25°C, and >26°C). In addition, each subgroup was jointly analyzed with regions to trace the source of heterogeneity. The heterogeneity of the covariates is explained in *R*^2^.

## Results

### Search Results and Qualification Studies

**Figure 1:**
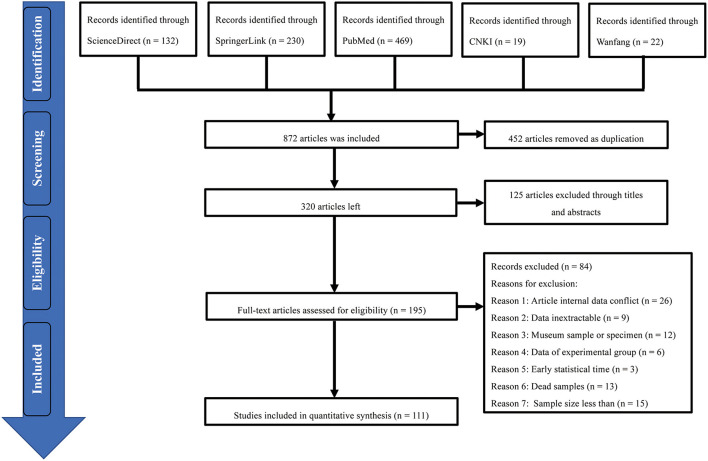
Flow diagram of the study screening process of inclusion and exclusion of studies.

According to the inclusion criteria for each article, we finally selected 111 studies from the five databases for meta-analysis (Figure 1), including 20 publications of medium quality (2–3 points) and 91 publications of high quality (4 or 5 points; Supplementary Table 4).

### Publication Bias and Sensitivity Analysis

**Figure 2:**
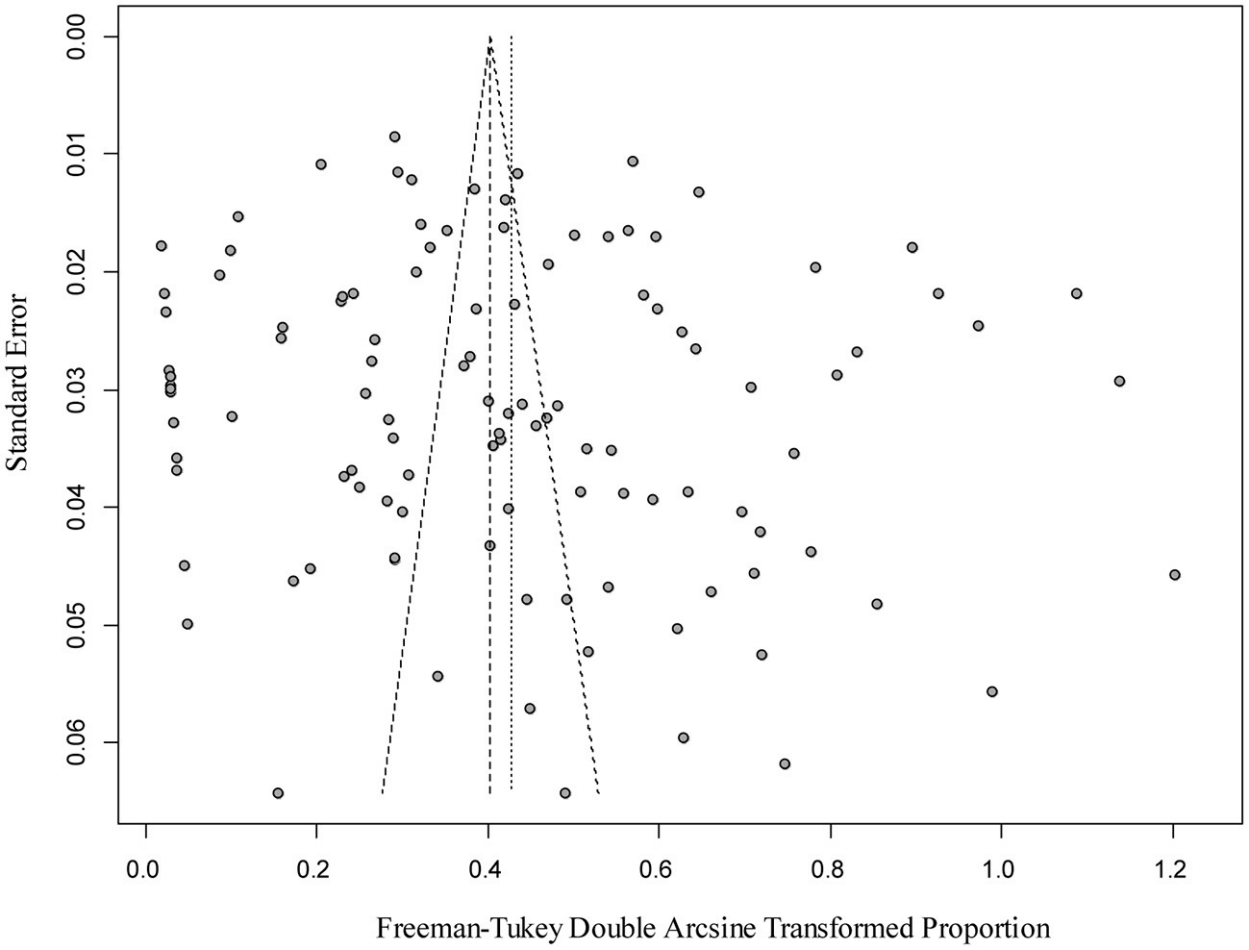
Funnel plot of the analysis publications bias of studies.

The results of our funnel plots could not directly judge whether there was publication bias in the included studies (Figure 2), but the result of an Egger's test demonstrated that there was no publication bias (*P* > 0.05; Supplementary Table 2). A forest plot illustrated the prevalence estimates of *Bd* in amphibians worldwide, showing an obvious heterogeneity between studies (χ^2^ = 11, 374.69, P = 0; I^2^ = 99.00%, 95% CI: 13.76–20.52; Supplementary Figure 1). Finally, the sensitivity analysis clarified the data reliability, when excluding any one study, the estimate values laid within the 95% CI of the respective overall prevalence, and had no significant influence (Supplementary Figure 2).

### Results of Meta-Analysis

**Figure 3:**
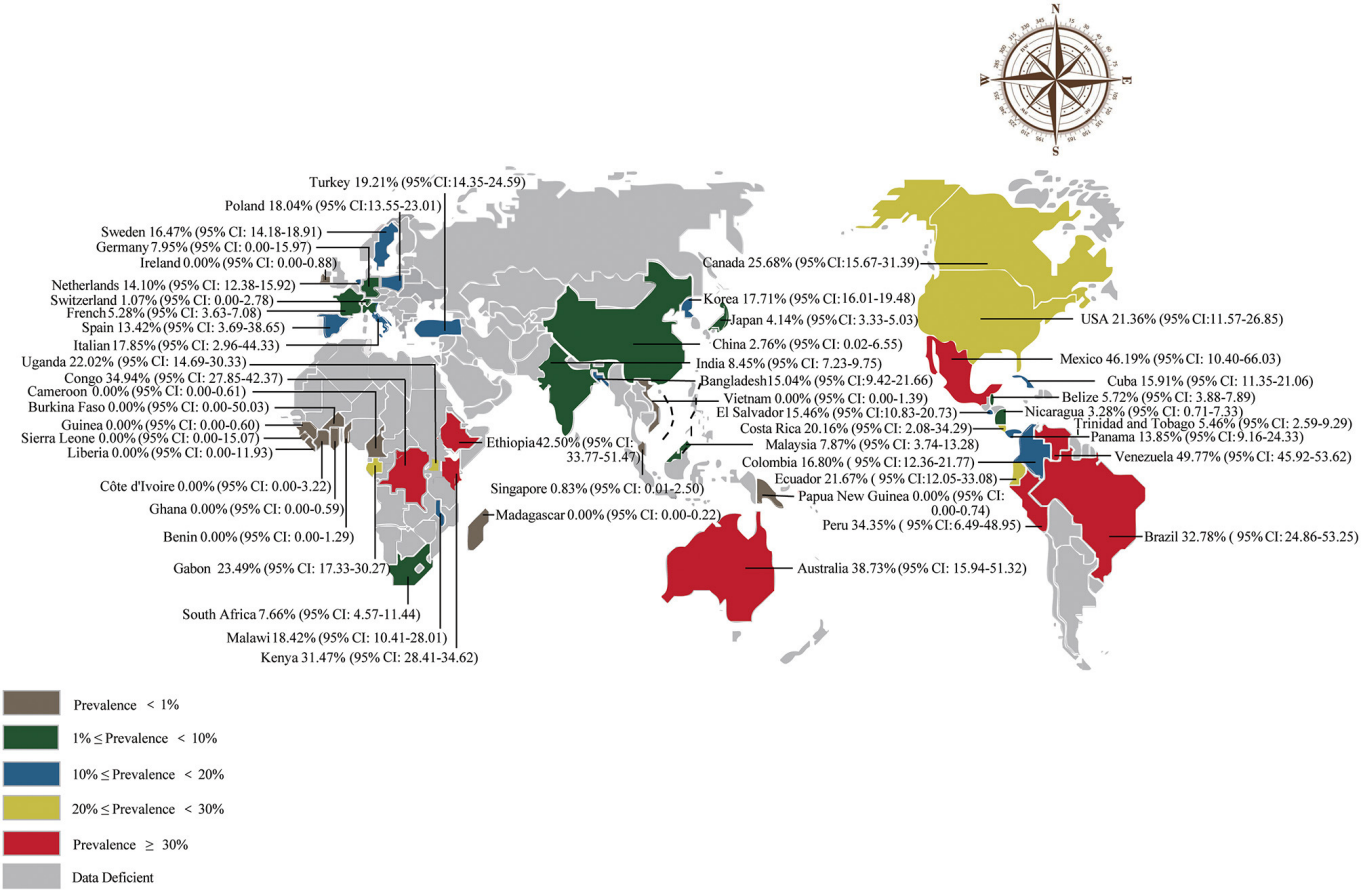
Map of the prevalence of *Batrachochytrium dendrobatidis* in amphibians worldwide.

A total of 50,985 amphibians were surveyed, and the prevalence of *Bd* in amphibians worldwide was 18.54% (95% CI: 13.76–20.52; Supplementary Table 3). The detailed *Bd* prevalence in amphibians from different regions ranged from 7.88% (95% CI: 1.92–8.71) to 36.34% (95% CI: 11.31–46.52; Table 1). The prevalence was lower in Asia than in other continents (*P* < 0.05; Table 1). Furthermore, the prevalence of *Bd* varied among countries (Supplementary Table 3). Venezuela had the highest prevalence at 49.77% (95% CI: 45.92–53.62), and the lowest prevalence was Benin, Burkina Faso, Cameroon, Côte d'Ivoire, Ghana, Guinea, Liberia, Madagascar, Papua New Guinea, Ireland, Sierra Leone and Vietnam at 0.00% (Supplementary Table 3; Figure 3).

**Table 1:** Pooled worldwide prevalence of *Batrachochytrium dendrobatidis* by region.

**Factor**	**Category**	**No. studies**	**No. tested**	**No. positive**	Prevalence (%) (95% CI^*^)	**Heterogeneity**			**Univariate meta-regression**		Joint analysis^*^
						**χ^2^**	***P*-value**	***I*^2^ (%)**	***P*-value**	**Coefficient (95% CI)**	R^2*^-region
Region									0.0007	−0.2246 (−0.3546 to −0.0946)	18.04%
	Africa	11	3,634	475	13.07% (2.49–24.25)	1083.42	<0.01	99.10%			
	Asia	14	9,113	718	7.88% (1.92–8.71)	671.83	<0.01	98.10%			
	Europe	15	9,038	976	10.80% (6.34–14.39)	481.09	<0.01	97.10%			
	North America	53	21,767	4,623	21.24% (14.20–24.16)	4573.64	0	98.90%			
	Oceania	10	3,767	1,369	36.34% (11.31–46.52)	1398.02	<0.01	99.40%			
	South America	11	3,666	1,291	35.21% (24.89–41.92)	258.85	<0.01	96.10%			
Sampling years									0.6350	0.0220 (−0.0687 to 0.1126)	14.55%
	2000–2008	50	18,884	3,851	20.39% (13.33–25.30)	5388.99	0	99.10%			
	After 2009	65	27,369	5,176	18.91% (13.23–21.56)	5229.69	0	98.80%			
Disease dynamic									<0.001	0.4853 (0.2689–0.7017)	23.05%
	Enzootic	71	35,533	5,729	16.12% (10.50–17.95)	7092.52	0	99.00%			
	Epizootic	5	1,064	312	29.32% (32.13–84.63)	126.42	<0.01	96.80%			
Detection method									0.0045	−0.2823 (−0.4769 to −0.0877)	20.48%
	Conventional PCR	19	9,414	1,721	18.28% (12.30–28.32)	1531.27	<0.01	98.80%			
	Nested PCR	7	5,595	703	12.56% (11.46–34.35)	567.85	<0.01	98.90%			
	Real-time PCR	84	34,828	7,003	20.11% (13.12–21.38)	8779.72	0	99.10%			
	Histopathology examination	6	2,254	77	3.42% (0.05–6.61)	141.03	<0.01	96.50%			
	LAMP^*^	1	456	0	0.00% (0.00–0.38)	0.00	–	–			
Host									0.0407	0.0793 (0.0034–0.1553)	15.21%
	Caecilian	3	9	0	0.00% (0.00–17.89)	0.24	0.89	0.00%			
	Frog	88	33,010	6,615	20.04% (13.52–21.71)	8199.42	0	98.90%			
	Salamander	31	4,603	642	13.95% (2.36–16.86)	1575.65	<0.01	98.10%			
	Toad	45	8,164	1,256	15.38% (6.16–14.92)	1144.52	<0.01	96.20%			
	Newt	10	1,270	208	16.38% (1.84–19.08)	115.82	<0.01	92.20%			
Ages									0.0528	−0.1396 (−0.2809 to 0.017)	20.66%
	Adult	50	18,492	4,487	24.26% (16.43–28.75)	4484.73	0	98.90%			
	Subadult	5	336	30	8.93% (5.23–71.43)	66.47	<0.01	94.00%			
	Juvenile	13	1,897	807	42.54% (17.68–49.64)	519.63	<0.01	97.70%			
	Tadpole	7	2,898	343	11.84% (4.71–25.62)	1028.73	<0.01	98.30%			
Season									0.1249	0.0998 (−0.0277 to 0.2273)	10.80%
	Spring	33	6,057	1,264	20.87% (14.78–31.62)	1879.08	0	98.30%			
	Summer	39	8,831	1,754	19.86% (8.51–22.97)	2720.77	0	98.60%			
	Autumn	10	1,001	99	9.89% (3.48–26.91)	198.08	<0.01	95.50%			
	Winter	17	2,725	453	16.62% (1.72–26.41)	1484.78	<0.01	98.90%			
IUCN category									0.4707	0.0703 (−0.1206 to 0.2612)	15.51%
	Least concern	86	32,033	5,225	16.31% (11.52–18.72)	6682.75	0	98.70%			
	Near threatened	27	2,185	373	17.07% (9.78–28.53)	445.02	<0.01	94.20%			
	Vulnerable	24	1,323	570	43.08% (3.25–36.86)	527.76	<0.01	95.60%			
	Endangered	28	2,040	486	23.82% (4.94–24.54)	636.66	<0.01	95.80%			
	Critically endangered	9	467	133	28.48% (0.00–57.62)	383.99	<0.01	97.90%			
Sample source									0.0920	−0.1985 (−0.4294 to 0.0324)	0.00%
	Forest	15	2,900	597	20.59% (3.92–33.53)	1461.70	<0.01	99.00%			
	Park	7	728	111	15.25% (0.00–12.87)	92.09	<0.01	93.50%			
	Lake	4	569	89	15.64% (7.95–34.77)	43.08	0.04	93.00%			
	Pond	21	4,975	913	18.35% (11.31–23.12)	497.39	<0.01	96.00%			
	Stream	22	3,960	915	23.11% (11.05–36.73)	1519.28	<0.01	98.60%			
	Others^*^	5	384	85	22.13% (0.51–39.93)	105.69	<0.01	96.20%			
Quality level									0.0847	0.1010 (−0.0138 to 0.2159)	18.66%
	Middle	20	7,765	808	10.41% 6.55–16.59)	895.50	<0.01	97.90%			
	High	91	43,220	9,644	20.00% (14.65–22.50)	10020.32	0	99.10%			
Total		111	50,985	9,452	18.54% (13.76–20.52)	11374.69	0	99.00%			

CI^*^ Confidence interval.

Joint analysis^*^ with prevalence of regions in worldwide;

R^2*^: Proportion of between-study variance explained.

LAMP^*^, Loop-mediated isothermal amplification.

Others^*^, Wetland (2), Road (3).

We next estimated the possible potential risk factors for *Bd* prevalence, including geographical distribution, sampling year, detection methods, host, season, age, IUCN category and sample source (Table 1). The geographical distribution, disease dynamic, detection method and host were identified as main potential risk factors for *Bd* prevalence (*P* < 0.05). The pooled prevalence of *Bd* in the 2000–2008 group was 20.39% (95% CI: 13.33–25.30), which was higher than 18.91% (95% CI: 13.23–21.56) in the after 2009 group (Table 1). The epizootic disease had a significantly higher prevalence of 29.32% (95% CI: 32.13–84.63) relative to enzootic disease. Between real-time PCR, nested PCR, conventional PCR, histopathology examination and LAMP, the first method detected the highest prevalence at 20.11% (95% CI: 13.12–21.38). However, the frogs had the highest prevalence of 20.04% (95% CI: 13.52–21.71) relative to other groups. The prevalence of *Bd* in juvenile amphibians was higher at 42.54% (95% CI: 17.68–49.64) than the prevalence in other ages. Meanwhile, we found that the prevalence in spring was higher at 20.87% (95% CI: 14.78–31.62) than in other seasons. The prevalence in vulnerable amphibians was 43.08% (95% CI: 3.25–36.86) and was higher than in other categories. However, the prevalence from stream samples was higher at 23.11% (95% CI: 11.05–36.73) than in other sample sources.

Following this, we analyzed the geographical factors subgroup factors including latitude, longitude, elevation, monthly average rainfall and monthly average temperature (Table 2). The *Bd* prevalence in the south latitude range (20–40°; 32.38%, 95% CI: 16.83–40.35) and in the west longitude range (100–160°; 31.81%, 95% CI: 14.17–41.80) was significantly higher than other latitude and longitude ranges (*P* < 0.05). In the 1,700–5,000 m elevation, the prevalence was 33.15% (95% CI: 9.64–34.27), which was significantly higher than other elevation ranges (*P* < 0.05). The monthly average rainfall in the over 150 mm precipitation range had the highest prevalence at 28.58% (95% CI: 12.69–30.42). The prevalence in the monthly average temperature range of 17–25°C reached 26.48% (95% CI: 18.18–38.13), which was significantly higher than other temperature ranges (*P* < 0.05). The heterogeneity of each subgroup was explained by region (the covariate), ranging from 0 to 30.53% (*R*^2^-region) (Tables 1, 2).

**Table 2:** Estimated pooled prevalence of *Batrachochytrium dendrobatidis* by geographical factor.

**Factor**	**Category**	**No. studies**	**No. tested**	**No. positive**	Prevalence (%) (95% CI*)	**Heterogeneity**			**Univariate meta-regression**		**Joint analysis**
						***χ*** ^2^	***P*** **-value**	***I***^2^ **(%)**	***P*** **-value**	**Coefficient (95% CI)**	**R^2^-region**
Elevation									0.0429	−0.1033 (−0.2033 to −0.0033)	30.53%
	0–100 m	39	8,226	864	10.50% (5.75–12.12)	772.68	<0.01	95.10%			
	100–500 m	38	7,613	956	12.56% (6.59–16.58)	1412.29	<0.01	97.40%			
	500–1700 m	56	10,006	2,570	25.68% (10.46–23.85)	3939.94	0	98.60%			
	1700–5000 m	23	2,742	909	33.15% (9.64–34.27)	1155.42	<0.01	98.10%			
Latitude									0.0206	0.1571 (0.0241–0.2902)	12.19%
	0–15°N	24	9,626	1,528	15.87% (7.77–20.09)	1774.75	0	98.70%			
	0–20°S	12	2,431	555	22.83% (5.92–42.19)	1249.07	<0.01	99.10%			
	15–30°N	17	4,014	858	21.38% (5.84–30.56)	1830.32	0	99.10%			
	20–40°S	15	4,367	1,414	32.38% (16.83–40.35)	1059.28	<0.01	98.70%			
	30–60°N	44	21,109	3,051	14.45% (9.96–18.74)	3470.40	0	98.80%			
Longitude									0.0193	0.1562 (0.0253–0.2871)	15.59%
	0–80°E	22	10,019	1,184	11.82% (6.00–15.76)	1197.09	<0.01	98.20%			
	0–100°W	52	17,178	2,993	17.42% (11.98–21.10)	3241.46	0	98.40%			
	80–120°E	11	2,299	185	8.05% (1.41–16.36)	450.51	<0.01	97.80%			
	100–160°W	16	4,049	1,288	31.81% (14.17–41.80)	1474.84	<0.01	99.00%			
	120–160°E	15	8,515	1,764	20.72% (6.66–31.04)	2945.45	0	99.50%			
Rainfall									0.9431	−0.0042 (−0.1191 to 0.1107)	3.47%
	0–50 mm	16	3,798	702	18.48% 13.60–30.72)	562.71	<0.01	97.30%			
	50–100 mm	19	5,855	1,282	21.90% (12.29–31.28)	980.61	<0.01	98.20%			
	100–150 mm	20	3,606	673	18.66% (13.05–30.37)	665.95	<0.01	97.10%			
	>150 mm	21	6,336	1,811	28.58% (12.69–30.42)	1295.02	<0.01	98.50%			
Temperature									0.0429	0.1288 (0.0041–0.2534)	0.00%
	5–16°C	25	6,025	1,211	20.10% (10.47–24.93)	1094.83	<0.01	97.80%			
	17–25°C	16	6,311	1,671	26.48% (18.18–38.13)	1899.14	0	98.70%			
	>26°C	15	2,814	450	15.99% (5.89–22.13)	406.34	<0.01	96.60%			

## Discussion

Chytridiomycosis is a worldwide infectious disease of amphibians, causing a decrease in biodiversity and a loss of economic benefits (42). Detailed knowledge of the epidemiological status of chytridiomycosis in amphibians provides a foundation for implementing efficient protective measures.

Therefore, we conducted a systematic review and meta-analysis of global *Bd* infection. The overall prevalence of *Bd* in amphibians worldwide from 2000 to 2021 was 18.54% (95% CI: 13.76–20.52; Supplementary Table 3).

In 2008, Office International des Epizooties (OIE) listed *Batrachochytrium dendrobatidis* as notifiable pathogen, because the international community recognized the impact of the global trade in amphibians on the spreading of chytridiomycosis (43). This legislation enabled countries connected to the World Trade Organization to specifically detect and restrict the trade of infected amphibians. Therefore, we chose 2008 as the entry point for our analysis of *Bd* infection in amphibians worldwide. We found that the *Bd* prevalence (18.91%) decreased after this policy was enacted (Table 1). Implementing similar policies might help to reduce *Bd* infection. Therefore, we suggest that more relevant legislation be implemented to protect amphibian species.

In our study, the *Bd* prevalence in South America and Oceania was basically the same and significantly higher than that in Asia (*P* < 0.05; Table 1). In view of our geographic subgroup analysis, we found that the highest *Bd* prevalence was in the latitude range 20–40°S and the longitude range 100–160°W (Table 2), which is also mainly concentrated in Oceania, Africa, South America and North America. Although *Bd* infection is mostly global, the distribution of fungus causing amphibian deaths has so far been limited to a few regions, mainly in Oceania, South America and North America (13, 44). Similarly, we found a high *Bd* prevalence in Australia, Brazil, Peru, Venezuela, Mexico, and the United States (21–49%; Supplementary Table 3). However, there were some areas where the prevalence of *Bd* was zero, mostly in Africa and Asia (Supplementary Table 3), which may be under-explored studies on the dynamics of amphibian chytridiomycosis (45). Or maybe frog populations in Africa have co-evolved with *Bd* to show resistance, even if the *Bd* was originated Africa (11, 46). When the elevation was between 1,700 and 5,000 m, the prevalence was significantly higher than that in the 0–100, 100–500, and 500–1,700 m groups (*P* < 0.05; Table 2). The joint analysis showed that regions explained 30.53% of heterogeneity in elevation subgroup (Table 2). These results may be due to the fact that different continents have different elevation characteristics. At high elevation areas, the prevalence of *Bd* increased and the survival rate of infected amphibians have been shown to be reduced (47, 48). In the entire Neotropical region, environmental conditions not only promote the abundance and diversity of amphibians, but also contribute to the growth and survival of *Bd*, especially in the cool and humid environments of many uplands (13). In central America, most amphibian declines occurred at elevations above 500 m (49). Since *Bd* is dependent on aquatic habitats (50) and intolerant to desiccation (51), higher levels of precipitation favor moist microhabitats and promote its growth and development (52, 53). The prevalence and infection intensity of chytridiomycosis are significantly correlated with temperature and rainfall in eastern Australia (54, 55). High environmental humidity may have promoted the growth of *Bd*, which is associated with high disease severity (56). In order to prevent the spread of *Bd* between various regions, we suggest that the domestic and international trade in amphibians should follow the veterinary supervision and quarantine guidelines issued by the IUCN and the OIE (43). According to the environmental conditions of specific locations and habitats, comprehensively evaluate the distribution, prevalence and infection intensity of *Bd*, and formulate more effective disease management strategies. In high-prevalence areas of *Bd* (more at risk compared to those in low-prevalence areas), more precautionary measures should be taken. These measures could include 1) closely stricter monitoring (increased intensity of supervisory personnel) the health of amphibians entering and exiting these areas and 2) continuously check the prevalence of *Bd* in amphibian habitats in these areas. If *Bd* is identified, fungicides could be used to control its spread (57).

For the seasonal subgroup, amphibians had the highest prevalence in spring (Table 1). The occurrence of chytridiomycosis has obvious seasonality (58). The fungal prevalence and infection intensity decreased from spring to summer within a population of frogs, which can clear fungal infections later in summer and at higher temperatures (59, 60). *Bd* is usually not randomly distributed in amphibian communities (47, 61), but rather is closely related to changes in microclimatic conditions. Therefore, we analyzed the most suitable conditions for *Bd* growth in combination with temperature and rainfall subgroups. Our results showed that amphibians were more susceptible to *Bd* infections at a temperature of 17–25°C (*P* < 0.05) and in rainfall of >150 mm (Table 2). On large geographic scales, temperatures and rainfall are the most fatal environmental factors affecting the occurrence of fungi, increasing the possibility of infection and transmission (10, 62). In view of the environmental scope of the above areas, we recommend multiple methods to control the spread of diseases such as *Bd*. Chemical methods are used in small epidemic areas, such as antifungal itraconazole, as well as environmental disinfectants (16, 63). Bio-augmentation methods have been proposed for more complex ecological environment, such as enhancing the amphibian cutaneous probiotic microorganisms (which can inhibit the growth of *Bd*) and vaccination to prevent fungal invasion (64).

In the 111 articles surveyed here, real-time PCR methods had a higher *Bd* prevalence (Table 1). Real-time PCR is more sensitive than traditional PCR and histopathology examination, with a higher and faster detection rate, making the results more objective rather than subjective (65). It can accurately detect *Bd* infection intensity in degraded samples or the low levels of organisms, increasing the detection of prevalence, and it is suitable for wildlife, laboratory animals, and the food trade as well as environmental samples (66). Therefore, most researchers prefer to use the real-time PCR method for the detection of *Bd* as early as possible. In addition, there are articles using nested PCR and Loop-mediated isothermal amplification (LAMP). Nested PCR and LAMP hhhimprove the sensitivity of traditional PCR, but are more prone to contamination (67, 68). Consequently, they have been rarely widely used. In fact, the use of appropriate detection methods is vital for helping relevant personnel obtain accurate testing data and better understand the epidemic status of a disease.

Among *Bd* infections in amphibians of different ages, the point estimate of juveniles was higher than the other three age ranges, but this was not significant (*P* = 0.0528; Table 1). The susceptibility to *Bd* was shown to vary at the different stages of life (61). The results have shown that the *Bd* prevalence in younger life stages was higher than in older life stages (larvae: 18.7%, juveniles: 40%, sub-adults: 5.5%; adults: 6.6%), and that juvenile stages had the highest prevalence of *Bd* (69). As frogs get older, *Bd* infection can be cleared (70). Fungi have mobile zoospores that have the ability to spread rapidly in water, which might promote tadpole infections (71), but tadpoles rarely died from these infections (51, 72). Thus, tadpoles might be the reservoirs for this disease and increase the activity of this *Bd* in the environment, leading to the infection of adult specimens (27, 73). Therefore, we recommend that relevant personnel pay attention to the detection *Bd* of tadpoles and juvenile amphibians, and control the fungal loads in environments where tadpoles live to reduce the prevalence.

Water sources can be one of the environmental reservoirs of *Bd*, because it can survive in water alone for several weeks (8, 71, 74). In a subgroup analysis of sample sources, the *Bd* prevalence of amphibians inhabiting streams was shown to be higher than in other places (Table 1). However, species living on roads had the lowest prevalence. Therefore, especially cooler and shaded lentic streams might be more conducive to *Bd* transmission among amphibians (75, 76). Thus, we analyzed the subgroups of different hosts, and frogs had the highest prevalence (*P* < 0.05; Table 1). There was a great difference in the degree of *Bd* infection between anurans (frogs and toads) and caudates (salamanders and newts) (72). According to statistical data, 36% of caudates are terrestrial, while only 1.7% of anurans are terrestrial (77). Therefore, species living longer in water were more susceptible to infection (78). In the light of the IUCN classification of amphibians, the prevalence of *Bd* in vulnerable species was the highest at 43.08% (Table 1). Many species were listed as threatened by the IUCN, because they were infected with *Bd* resulting in population declines (79). Therefore, we propose strengthening the protective measures for those habitats that are suitable for *Bd* growth, such as manipulating the habitat environment for temperature or humidity and for endangered species, captive breeding may be established to ensure populations.

In our research, there were 20 medium-quality articles and 91 high-quality articles (Table 1). In the medium quality articles, we found that some of them did not have random sampling, definite sampling time, sampling location or clear detection methods (Supplementary Table 4). Since these medium-quality articles did not provide detailed data, the potential risk factors of *Bd* infection cannot be accurately measured, leading to biased results. Thus, we recommend that researchers should find as many factors as possible influencing fungal infection, so as to more effectively prevent these diseases and lay scientific groundwork for future research work.

This study has many advantages, such as its rich sample size, comprehensive coverage, clear method of analyses and wide range of risk factor assessments, which provides many evidence-based substantive suggestions for combating *Bd* spread. However, this study has also some shortcomings. First, although corresponding search formula is applied to collect articles, all relevant studies could not be fully found. Second, there was no information about the susceptibility of a species to *Bd* infection, it could be a potentially important risk factor for the implementation of controls. Third, only 13 out of 111 studies included in the investigation reported explicitly using random sampling, but others could not be ruled out the possibility of random sampling, which could result in a slight publication bias in the funnel plots and Egger's test, even through no publication bias was detected in this study. Therefore, the future study design still need to be comprehensive, such as clearly disclosing their sampling details. However, we are convinced that our report can reflect the situation of chytridiomycosis spread worldwide.

## Conclusion

Chytridiomycosis has a wide distribution range and affects various amphibian species at all ages. In the last decade, the prevalence of *Bd* has shown a decreasing trend, and temperature and elevation have significantly affected it. In addition, the use of real-time PCR methods could help researchers obtain a more accurate estimate of *Bd* prevalence in amphibians. Meanwhile, we thus should focus on highly sensitive species and those listed as threatened by the IUCN. Therefore, we suggest that governments should formulate a reasonable management based on different geographical conditions, habitat types and species status. This study helps further elucidate potential risk factors for *Bd* infection and also provides a theoretical basis for related personnel to prevent and control chytridiomycosis transmission.

## Data Availability Statement

The original contributions presented in the study are included in the article/Supplementary Material, further inquiries can be directed to the corresponding authors.

## Author Contributions

ZL performed the data extraction and drafted the manuscript. ZL and QW analyzed the data. KS and JF developed the paper concept, designed, supervised, and revised the manuscript. All authors contributed to the article and approved the submitted version.

## Funding

This work was supported by the National Natural Science Foundation of China (grant numbers 31961123001, 32171525, and 31770403).

## Conflict of Interest

The authors declare that the research was conducted in the absence of any commercial or financial relationships that could be construed as a potential conflict of interest.

## Publisher's Note

All claims expressed in this article are solely those of the authors and do not necessarily represent those of their affiliated organizations, or those of the publisher, the editors and the reviewers. Any product that may be evaluated in this article, or claim that may be made by its manufacturer, is not guaranteed or endorsed by the publisher.
