# Supplementary material for: Prevalence of Batrachochytrium dendrobatidis in Amphibians From 2000 to 2021: A Global Systematic Review and Meta-Analysis
Source: Front Vet Sci. 2021 Dec 17;8:791237. doi: 10.3389/fvets.2021.791237 (PMC8718539; doi:10.3389/fvets.2021.791237)
Supplement: Supplementary file 1 [file Data_Sheet_1.docx]

**Prevalence of** ***Batrachochytrium dendrobatidis* in amphibians from 2000 to 2021: A global systematic review and meta-analysis**

Zhongle Li^1,2,a^, Qi Wang^2,a^, Keping Sun^1,3,*^, and Jiang Feng^1,2,*^

^1^ Jilin Provincial Key Laboratory of Animal Resource Conservation and Utilization, Northeast Normal University, 2555 Jingyue Street, Changchun 130117, China

^2^ College of Animal Science and Technology, Jilin Agricultural University, Changchun 130018, China

^3^ Key Laboratory of Vegetation Ecology, Ministry of Education, Changchun, 130024, China

* Correspondence to: sunkp129@nenu.edu.cn and fengj@nenu.edu.cn

^a^ These authors contributed equally to this work.

**Table S1** Normal distribution test for the normal rate and the different conversion of the normal rate.

| Conversion form | *W* | *P* |
| --- | --- | --- |
| PRAW | 0.87597 | 3.62e-08 |
| PLN | NaN | NA |
| PLOGIT | NaN | NA |
| PAS | 0.96851 | 0.009998 |
| PFT | 0.96961 | 0.01231 |

“*W*”: Shapiro-Wilk test; “PRAW”: original rate; “PLN”: logarithmic conversion; “PLOGIT”: logit transformation; “PAS”: arcsine transformation; “PFT”: double-arcsine transformation; “NaN”: meaningless number; “NA”: missing data.

**Table S2** Egger’s test for Publication Bias.

| slope | bias | se. bias | t | df | *p*-value |
| --- | --- | --- | --- | --- | --- |
| 0.357 | 2.232 | 2.142 | 1.042 | 109 | 0.2999 |

**Table S3** Estimated pooled prevalence of *Batrachochytrium dendrobatidis* by national region.

| Countries | No.studies | No.tested | No.positive | %Prevalence | % (95% CI) |
| --- | --- | --- | --- | --- | --- |
| Australia | 9 | 3535 | 1369 | 38.73% | 15.94-51.32 |
| Bangladesh | 1 | 133 | 20 | 15.04% | 9.42-21.66 |
| Belize | 1 | 524 | 30 | 5.72% | 3.88-7.89 |
| Benin | 1 | 133 | 0 | 0.00% | 0.00-1.29 |
| Brazil | 6 | 1138 | 373 | 32.78% | 24.86-53.25 |
| Burkina Faso | 1 | 3 | 0 | 0.00% | 0.00-50.03 |
| Cameroon | 1 | 283 | 0 | 0.00% | 0.00-0.61 |
| Canada | 5 | 3209 | 824 | 25.68% | 15.67-31.39 |
| China | 6 | 2425 | 67 | 2.76% | 0.02-6.55 |
| Colombia | 1 | 244 | 41 | 16.80% | 12.36-21.77 |
| Congo | 1 | 166 | 58 | 34.94% | 27.85-42.37 |
| Costa Rica | 6 | 1399 | 282 | 20.16% | 2.08-34.29 |
| Côte d'Ivoire | 1 | 53 | 0 | 0.00% | 0.00-3.22 |
| Cuba | 1 | 220 | 35 | 15.91% | 11.35-21.06 |
| Ecuador | 1 | 60 | 13 | 21.67% | 12.05-33.08 |
| El Salvador | 1 | 207 | 32 | 15.46% | 10.83-20.73 |
| Ethiopia | 1 | 120 | 51 | 42.50% | 33.77-51.47 |
| French | 2 | 663 | 35 | 5.28% | 3.63-7.08 |
| Gabon | 1 | 166 | 39 | 23.49% | 17.33-30.27 |
| Germany | 2 | 3570 | 284 | 7.95% | 0.00-15.97 |
| Ghana | 1 | 290 | 0 | 0.00% | 0.00-0.59 |
| Guinea | 1 | 287 | 0 | 0.00% | 0.00-0.60 |
| India | 1 | 1870 | 158 | 8.45% | 7.23-9.75 |
| Ireland | 1 | 195 | 0 | 0.00% | 0.00-0.88 |
| Italian | 4 | 762 | 136 | 17.85% | 2.96-44.33 |
| Japan | 1 | 2103 | 87 | 4.14% | 3.33-5.03 |
| Kenya | 1 | 861 | 271 | 31.47% | 28.41-34.62 |
| Korea | 1 | 1863 | 330 | 17.71% | 16.01-19.48 |
| Liberia | 1 | 14 | 0 | 0.00% | 0.00-11.93 |
| Madagascar | 2 | 827 | 0 | 0.00% | 0.00-0.22 |
| Malawi | 1 | 76 | 14 | 18.42% | 10.41-28.01 |
| Malaysia | 1 | 127 | 10 | 7.87% | 3.74-13.28 |
| Mexico | 6 | 1115 | 515 | 46.19% | 10.40-66.03 |
| Netherlands | 1 | 1482 | 209 | 14.10% | 12.38-15.92 |
| Nicaragua | 1 | 122 | 4 | 3.28% | 0.71-7.33 |
| Panama | 4 | 3560 | 493 | 13.85% | 9.16-24.33 |
| Papua New Guinea | 1 | 232 | 0 | 0.00% | 0.00-0.74 |
| Peru | 2 | 1575 | 541 | 34.35% | 6.49-48.95 |
| Poland | 1 | 255 | 46 | 18.04% | 13.55-23.01 |
| Sierra Leone | 1 | 11 | 0 | 0.00% | 0.00-15.07 |
| Singapore | 1 | 240 | 2 | 0.83% | 0.01-2.50 |
| South Africa | 1 | 235 | 18 | 7.66% | 4.57-11.44 |
| Spain | 2 | 790 | 106 | 13.42% | 3.69-38.65 |
| Sweden | 1 | 947 | 156 | 16.47% | 14.18-18.91 |
| Switzerland | 1 | 374 | 4 | 1.07% | 0.00-2.78 |
| Trinidad and Tobago | 1 | 183 | 10 | 5.46% | 2.57-9.29 |
| Turkey | 1 | 229 | 44 | 19.21% | 14.35-24.59 |
| Uganda | 1 | 109 | 24 | 22.02% | 14.69-30.33 |
| USA | 27 | 11228 | 2398 | 21.36% | 11.57-26.85 |
| Venezuela | 1 | 649 | 323 | 49.77% | 45.92-53.62 |
| Vietnam | 1 | 123 | 0 | 0.00% | 0.00-1.39 |
| Total | 111 | 50985 | 9452 | 18.54% | 13.76-20.52 |

**Table S4** Included studies and quality scores.

|  | **Reference ID** | **No.**  **tested** | **No.**  **positive** | **Prevalence** | **Study design** | **Detailed sampling method or not** | **Definite**  **random sampling or not** | **Definite sampling time or not** | **Clear detection method or not** | **Four or more risk factors or not** | **Score** | **Study Quality** |
| --- | --- | --- | --- | --- | --- | --- | --- | --- | --- | --- | --- | --- |
| 1 | Zhu (2015) | 456 | 0 | 0.0% | Cross sectional | N* | N | N | Y* | Y | 2 | middle |
| 2 | Gilbert et al. (2013) | 240 | 2 | 0.8% | Cross sectional | Y | N | N | Y | Y | 3 | middle |
| 3 | Jacinto-Maldonado et al. (2019) | 116 | 3 | 2.6% | Cross sectional | N | N | Y | Y | Y | 3 | middle |
| 4 | Woodhams et al. (2008) | 783 | 83 | 10.6% | Cross sectional | N | N | Y | Y | Y | 3 | middle |
| 5 | Murrieta-Galindo  et al. (2014) | 109 | 20 | 18.3% | Cross sectional | N | N | Y | Y | Y | 3 | middle |
| 6 | Kärvemo et al. (2018) | 947 | 156 | 16.5% | Cross sectional | N | N | Y | Y | Y | 3 | middle |
| 7 | Zumbado-Ulate et al. (2014) | 410 | 10 | 2.4% | Cross sectional | N | N | Y | Y | Y | 3 | middle |
| 8 | Spitzen-van der Sluijs et al. (2017) | 1482 | 209 | 14.1% | Cross sectional | N | N | Y | Y | Y | 3 | middle |
| 9 | Bell et al. (2011) | 166 | 39 | 23.5% | Cross sectional | N | N | Y | Y | Y | 3 | middle |
| 10 | Cádiz et al. (2018) | 220 | 35 | 15.9% | Cross sectional | N | N | Y | Y | Y | 3 | middle |
| 11 | Bresciano et al. (2015) | 60 | 13 | 21.7% | Cross sectional | Y | N | N | Y | Y | 3 | middle |
| 12 | Raffel et al. (2010) | 153 | 63 | 41.2% | Cross sectional | N | N | Y | Y | Y | 3 | middle |
| 13 | Bai (2012) | 183 | 0 | 0.0% | Cross sectional | N | N | Y | Y | Y | 3 | middle |
| 14 | Beard and O’Neill (2005) | 382 | 9 | 2.4% | Cross sectional | N | N | Y | Y | Y | 3 | middle |
| 15 | Kaiser and Pollinger (2012) | 524 | 30 | 5.7% | Cross sectional | N | N | Y | Y | Y | 3 | middle |
| 16 | Chatfield et al. (2012) | 98 | 33 | 33.7% | Cross sectional | Y | N | N | Y | Y | 3 | middle |
| 17 | Zhu et al. (2016) | 1073 | 12 | 1.1% | Cross sectional | N | N | Y | Y | Y | 3 | middle |
| 18 | Coutinho et al. (2015) | 107 | 61 | 57.0% | Cross sectional | Y | N | N | Y | Y | 3 | middle |
| 19 | Wei et al. (2010) | 180 | 16 | 8.9% | Cross sectional | N | N | Y | Y | Y | 3 | middle |
| 20 | Conradie et al. (2011b) | 76 | 14 | 18.4% | Cross sectional | N | N | Y | Y | Y | 3 | middle |
| 21 | Ocock et al. (2013) | 271 | 17 | 6.3% | Cross sectional | Y | N | Y | Y | Y | 4 | high |
| 22 | Ribeiro et al. (2020) | 339 | 46 | 13.6% | Cross sectional | Y | N | Y | Y | Y | 4 | high |
| 23 | Nava-González et al. (2019) | 412 | 282 | 68.4% | Cross sectional | Y | N | Y | Y | Y | 4 | high |
| 24 | Flechas et al. (2012) | 244 | 41 | 16.8% | Cross sectional | Y | N | Y | Y | Y | 4 | high |
| 25 | Muletz-Wolz et al. (2018) | 100 | 0 | 0.0% | Cross sectional | Y | N | N | Y | Y | 3 | middle |
| 26 | Saenz et al. (2014) | 130 | 64 | 49.2% | Cross sectional | Y | N | Y | Y | Y | 4 | high |
| 27 | Savage et al. (2011) | 127 | 10 | 7.9% | Cross sectional | Y | N | Y | Y | Y | 4 | high |
| 28 | Petersen et al. (2016) | 670 | 137 | 20.4% | Cross sectional | Y | N | Y | Y | Y | 4 | high |
| 29 | Schlaepfer et al. (2007) | 112 | 42 | 37.5% | Cross sectional | Y | N | Y | Y | Y | 4 | high |
| 30 | Ernetti et al. (2020) | 65 | 30 | 46.1% | Cross sectional | Y | N | Y | Y | Y | 4 | high |
| 31 | Van Sluys and Hero (2010) | 280 | 118 | 42.1% | Cross sectional | Y | N | Y | Y | Y | 4 | high |
| 32 | Zumbado-Ulate et al. (2019) | 348 | 190 | 54.6% | Cross sectional | Y | N | Y | Y | Y | 4 | high |
| 33 | Rodríguez-Brenes et al. (2016) | 1695 | 159 | 9.4% | Cross sectional | Y | N | Y | Y | Y | 4 | high |
| 34 | Voordouw et al. (2010) | 320 | 42 | 13.1% | Cross sectional | Y | N | Y | Y | Y | 4 | high |
| 35 | Mendoza-Almeralla et al. (2016) | 160 | 12 | 7.5% | Cross sectional | Y | N | Y | Y | Y | 4 | high |
| 36 | Oficialdegui et al. (2019) | 165 | 46 | 27..9% | Cross sectional | Y | N | Y | Y | Y | 4 | high |
| 37 | Kilburn et al. (2011) | 879 | 202 | 23.0% | Cross sectional | Y | N | Y | Y | Y | 4 | high |
| 38 | Muletz et al. (2014) | 606 | 4 | 0.7% | Cross sectional | Y | N | Y | Y | Y | 4 | high |
| 39 | Bai et al. (2010) | 259 | 39 | 15.1% | Cross sectional | Y | N | Y | Y | Y | 4 | high |
| 40 | Goka et al. (2009) | 2103 | 87 | 4.1% | Cross sectional | Y | N | Y | Y | Y | 4 | high |
| 41 | Bataille et al. (2013) | 1863 | 330 | 17.7% | Cross sectional | Y | N | Y | Y | Y | 4 | high |
| 42 | Whitfield et al. (2013) | 253 | 54 | 21.3% | Cross sectional | Y | N | Y | Y | Y | 4 | high |
| 43 | Picco and Collins (2007) | 84 | 9 | 10.7% | Cross sectional | N | Y | Y | Y | Y | 4 | high |
| 44 | Goldberg et al. (2009) | 178 | 9 | 5.1% | Cross sectional | Y | N | Y | Y | Y | 4 | high |
| 45 | Saenz et al. (2009) | 126 | 10 | 7.9% | Cross sectional | Y | N | Y | Y | Y | 4 | high |
| 46 | Sánchez et al. (2008) | 649 | 323 | 49.8% | Cross sectional | Y | N | Y | Y | Y | 4 | high |
| 47 | Lannoo et al. (2011) | 1306 | 217 | 16.6% | Cross sectional | Y | N | Y | Y | Y | 4 | high |
| 48 | Puschendorf et al. (2011) | 291 | 240 | 82.5% | Cross sectional | Y | N | Y | Y | Y | 4 | high |
| 49 | Kriger et al. (2007) | 863 | 228 | 26.4% | Cross sectional | Y | N | Y | Y | Y | 4 | high |
| 50 | Stark et al. (2017) | 122 | 4 | 3.3% | Cross sectional | N | Y | Y | Y | Y | 4 | high |
| 51 | Lawson et al. (2011) | 207 | 32 | 15.5% | Cross sectional | Y | N | Y | Y | Y | 4 | high |
| 52 | Kriger and Hero. (2007) | 519 | 157 | 30.2% | Cross sectional | Y | N | Y | Y | Y | 4 | high |
| 53 | McMillan et al. (2020) | 2223 | 644 | 29.0% | Cross sectional | Y | N | Y | Y | Y | 4 | high |
| 54 | Sette et al. (2020) | 328 | 22 | 6.7% | Cross sectional | Y | N | Y | Y | Y | 4 | high |
| 55 | Kriger et al. (2006) | 467 | 148 | 31.7% | Cross sectional | Y | N | Y | Y | Y | 4 | high |
| 56 | Peralta-García et al. (2018) | 199 | 94 | 47.2% | Cross sectional | Y | N | Y | Y | Y | 4 | high |
| 57 | Hidalgo-Vila et al. (2012) | 625 | 60 | 9.6% | Cross sectional | Y | N | Y | Y | Y | 4 | high |
| 58 | Ohst et al. (2013) | 3450 | 284 | 8.2% | Cross sectional | Y | N | Y | Y | Y | 4 | high |
| 59 | Longo et al. (2010) | 525 | 336 | 64.0% | Cross sectional | Y | N | Y | Y | Y | 4 | high |
| 60 | Forzán et al. (2010) | 114 | 30 | 26.3% | Cross sectional | Y | N | Y | Y | Y | 4 | high |
| 61 | Goldberg et al. (2007) | 109 | 24 | 22.0% | Cross sectional | Y | N | Y | Y | Y | 4 | high |
| 62 | Gründler et al. (2012) | 396 | 136 | 34.3% | Cross sectional | Y | N | Y | Y | Y | 4 | high |
| 63 | Terrell et al. (2014) | 212 | 34 | 16.0% | Cross sectional | N | Y | Y | Y | Y | 4 | high |
| 64 | Vredenburg et al. (2012) | 300 | 0 | 0.0% | Cross sectional | Y | N | Y | Y | Y | 4 | high |
| 65 | Weldon et al. (2018) | 527 | 0 | 0.0% | Cross sectional | Y | N | Y | Y | Y | 4 | high |
| 66 | Pilliod et al. (2010) | 353 | 127 | 36.0% | Cross sectional | Y | N | Y | Y | Y | 4 | high |
| 67 | Rahman et al. (2021) | 133 | 20 | 15.0% | Cross sectional | Y | N | Y | Y | Y | 4 | high |
| 68 | Kolenda et al. (2017) | 255 | 46 | 18.0% | Cross sectional | Y | N | Y | Y | Y | 4 | high |
| 69 | Frías-Alvarez et al. (2018) | 119 | 104 | 87.4% | Cross sectional | Y | N | Y | Y | Y | 4 | high |
| 70 | Rebollar et al. (2014) | 203 | 49 | 24.1% | Cross sectional | Y | N | Y | Y | Y | 4 | high |
| 71 | Richards-Hrdlicka et al. (2013) | 916 | 261 | 28.5% | Cross sectional | N | Y | Y | Y | Y | 4 | high |
| 72 | Rowley et al. (2007) | 274 | 0 | 0.0% | Cross sectional | Y | N | Y | Y | Y | 4 | high |
| 73 | Mutnale et al. (2018) | 1870 | 158 | 8.4% | Cross sectional | Y | N | Y | Y | Y | 4 | high |
| 74 | Bales et al. (2015) | 91 | 22 | 24.2% | Cross sectional | Y | N | Y | Y | Y | 4 | high |
| 75 | Jaeger et al. (2017) | 376 | 26 | 6.9% | Cross sectional | Y | N | Y | Y | Y | 4 | high |
| 76 | Rivera et al. (2019) | 215 | 17 | 7.9% | Cross sectional | Y | N | Y | Y | Y | 4 | high |
| 77 | Labumbard et al. (2020) | 1444 | 523 | 36.2% | Cross sectional | Y | N | Y | Y | Y | 4 | high |
| 78 | Zampiglia et al. (2013) | 469 | 66 | 14.1% | Cross sectional | N | Y | Y | Y | Y | 4 | high |
| 79 | Hunter et al. (2010) | 237 | 48 | 20.2% | Cross sectional | Y | N | Y | Y | Y | 4 | high |
| 80 | Erismis et al. (2014) | 229 | 44 | 19.2% | Cross sectional | Y | N | Y | Y | Y | 4 | high |
| 81 | Hardman et al. (2020) | 202 | 54 | 26.7% | Cross sectional | Y | N | Y | Y | Y | 4 | high |
| 82 | Keitzer et al. (2011) | 278 | 0 | 0.0% | Cross sectional | Y | N | Y | Y | Y | 4 | high |
| 83 | Group et al. (2016) | 155 | 26 | 16.8% | Cross sectional | Y | N | Y | Y | Y | 4 | high |
| 84 | Valencia-Aguilar et al. (2015) | 90 | 39 | 43.3% | Cross sectional | Y | N | Y | Y | Y | 4 | high |
| 85 | Aoust-Messier et al. (2015) | 482 | 84 | 17.4% | Cross sectional | Y | N | Y | Y | Y | 4 | high |
| 86 | Canestrelli et al. (2013) | 80 | 56 | 70.0% | Cross sectional | Y | N | Y | Y | Y | 4 | high |
| 87 | Federici et al. (2008) | 153 | 13 | 8.5% | Cross sectional | Y | N | Y | Y | Y | 4 | high |
| 88 | Ficetola et al. (2011) | 60 | 1 | 1.7% | Cross sectional | Y | N | Y | Y | Y | 4 | high |
| 89 | Kielgast et al. (2010) | 861 | 271 | 31.5% | Cross sectional | Y | N | Y | Y | Y | 4 | high |
| 90 | Brannelly et al. (2017) | 526 | 413 | 78.5% | Cross sectional | Y | N | Y | Y | Y | 4 | high |
| 91 | Penner et al. (2013) | 791 | 0 | 0.0% | Cross sectional | Y | N | Y | Y | Y | 4 | high |
| 92 | Gower et al. (2012) | 120 | 51 | 42.5% | Cross sectional | Y | N | Y | Y | Y | 4 | high |
| 93 | Bower et al. (2020) | 232 | 0 | 0.0% | Cross sectional | Y | N | Y | Y | Y | 4 | high |
| 94 | Lötters et al. (2012) | 310 | 0 | 0.0% | Cross sectional | Y | N | Y | Y | Y | 4 | high |
| 95 | Courtois et al. (2012) | 170 | 10 | 5.9% | Cross sectional | Y | N | Y | Y | Y | 4 | high |
| 96 | Doherty-Bone et al. (2008) | 283 | 0 | 0.0% | Cross sectional | Y | N | Y | Y | Y | 4 | high |
| 97 | Alemu et al. (2013) | 183 | 10 | 5.5% | Cross sectional | Y | N | Y | Y | Y | 4 | high |
| 98 | Thien et al. (2013) | 123 | 0 | 0.0% | Cross sectional | Y | N | Y | Y | Y | 4 | high |
| 99 | Ouellet et al. (2012) | 493 | 25 | 5.1% | Cross sectional | Y | N | Y | Y | Y | 4 | high |
| 100 | Slough (2009) | 70 | 24 | 34.3% | Cross sectional | Y | N | Y | Y | Y | 4 | high |
| 101 | Forrest et al. (2015) | 302 | 158 | 52.3% | Cross sectional | Y | N | Y | Y | Y | 4 | high |
| 102 | Harner et al. (2013) | 161 | 50 | 31.1% | Cross sectional | Y | N | Y | Y | Y | 4 | high |
| 103 | Gandola and Hendry (2013) | 195 | 0 | 0.0% | Cross sectional | Y | N | Y | Y | Y | 4 | high |
| 104 | Parrott et al. (2016) | 509 | 26 | 5.1% | Cross sectional | Y | Y | Y | Y | Y | 5 | high |
| 105 | Ruggeri et al. (2017) | 141 | 61 | 70.4% | Cross sectional | Y | Y | Y | Y | Y | 5 | high |
| 106 | Pearl et al. (2009) | 784 | 478 | 61.0% | Cross sectional | Y | Y | Y | Y | Y | 5 | high |
| 107 | Pullen et al. (2010) | 916 | 109 | 11.9% | Cross sectional | Y | Y | Y | Y | Y | 5 | high |
| 108 | Love et al. (2016) | 988 | 98 | 9.9% | Cross sectional | Y | Y | Y | Y | Y | 5 | high |
| 109 | Conradie et al. (2011a) | 235 | 18 | 7.7% | Cross sectional | Y | Y | Y | Y | Y | 5 | high |
| 110 | Hossack et al. (2010) | 756 | 7 | 0.9% | Cross sectional | Y | Y | Y | Y | Y | 5 | high |
| 111 | Greenbaum et al. (2015) | 166 | 58 | 34.9% | Cross sectional | Y | Y | Y | Y | Y | 5 | high |

Y*: Yes; N*: No.

1. Zhu, Y.Q. (2015). Detection of amphibian chytrid fungus and pest risk analysis (PRA) in China. *Northeast Forestry University*. (In Chinese)
2. Gilbert, M., Bickford, D., Clark, L., Johnson, A., Joyner, P.H., Ogg Keatts, L., Khammavong, K., Nguyễn Văn, L., Newton, A., Seow, T.P., Roberton, S., Silithammavong, S., Singhalath, S., Yang, A., Seimon, T.A. (2012). Amphibian pathogens in southeast Asian frog trade. *EcoHealth*, *9*(4), 386-398.
3. Jacinto-Maldonado, M., García-Peña, G.E., Paredes-León, R., Saucedo, B., Sarmiento-Silva, R.E., García, A., Martínez-Gómez, D., Ojeda, M., Del Callejo, E., Suzán, G. (2019). Chiggers (Acariformes: Trombiculoidea) do not increase rates of infection by *Batrachochytrium dendrobatidis* fungus in the endemic Dwarf Mexican Treefrog *Tlalocohyla smithii* (Anura: Hylidae). *International Journal for Parasitology-Parasites and Wildlife*, *11*, 163-173.
4. Woodhams, D.C., Kilburn, V.L., Reinert, L.K., Voyles, J., Medina, D., Ibáñez, R., Hyatt, A.D., Boyle, D.G., Pask, J.D., Green, D.M., Rollins-Smith, L.A. (2008). Chytridiomycosis and amphibian population declines continue to spread eastward in Panama. *EcoHealth*, *5*(3), 268-274.
5. Murrieta-Galindo, R., Parra-Olea, G., González-Romero, A., López-Barrera, F., Vredenburg, V.T. (2014). Detection of *Batrachochytrium dendrobatidis* in amphibians inhabiting cloud forests and coffee agroecosystems in central Veracruz, Mexico. *European Journal of Wildlife Research*, *60*(3), 431-439.
6. Kärvemo, S., Meurling, S., Berger, D., Höglund, J., Laurila, A. (2018). Effects of host species and environmental factors on the prevalence of *Batrachochytrium dendrobatidis* in northern Europe. *PLoS One*, *13*(10), e0199852.
7. Zumbado-Ulate, H., Bolaños, F., Gutiérrez-Espeleta, G., Puschendorf, R. (2014). Extremely low prevalence of *Batrachochytrium dendrobatidis* in frog populations from Neotropical dry forest of Costa Rica supports the existence of a climatic refuge from disease. *EcoHealth*, *11*(4), 593-602.
8. Spitzen-van der Sluijs, A., Canessa, S., Martel, A., Pasmans, F. (2017). Fragile coexistence of a global chytrid pathogen with amphibian populations is mediated by environment and demography. *Philosophical Transactions of the Royal Society B-Biological Sciences*, *284*(1864), 20171444.
9. Bell, R.C., Gata Garcia, A.V., Stuart, B.L., Zamudio, K.R. (2011). High prevalence of the amphibian chytrid pathogen in Gabon. *Ecohealth*, *8*(1), 116-120.
10. Cádiz, A., Reytor, M.L., Díaz, L.M., Chestnut, T., Burns, J.A., Amato, G. (2019). The chytrid fungus, *Batrachochytrium dendrobatidis*, is widespread among Cuban amphibians. *EcoHealth*, *16*(1), 128-140.
11. Bresciano, J.C., Salvador, C.A., Paz-Y-Miño, C., Parody-Merino, A.M., Bosch, J., Woodhams, D.C. (2015). Variation in the presence of anti-*Batrachochytrium dendrobatidis* bacteria of amphibians across life stages and elevations in Ecuador. *EcoHealth*, *12*(2), 310-319.
12. Raffel, T.R., Michel, P.J., Sites, E.W., Rohr, J.R. (2010). What drives chytrid infections in newt populations? Associations with substrate, temperature, and shade. *EcoHealth*, *7*(4), 526-536.
13. Bai, S.Z. (2012). Risk identification and characterization for *Batrachochytrium dendrobatidis* in anura in Changbai Mountain natural reserve. *Northeast Forestry University*. (In Chinese)
14. Beard, K.H., O’Neill, E.M. (2005). Infection of an invasive frog *Eleutherodactylus coqui* by the chytrid fungus *Batrachochytrium dendrobatidis* in Hawaii. *Biological Conservation*, *126*(4), 591-595.
15. Kaiser, K., Pollinger, J. (2012). *Batrachochytrium dendrobatidis* shows high genetic diversity and ecological niche specificity among haplotypes in the Maya Mountains of Belize. *PLoS One*, *7*(2), e32113.
16. Chatfield, M.W., Moler, P., Richards-Zawacki, C.L. (2012). The amphibian chytrid fungus, *Batrachochytrium dendrobatidis*, in fully aquatic salamanders from Southeastern North America. *PLoS One*, *7*(9), e44821.
17. Zhu, W., Fan, L., Soto-Azat, C., Yan, S., Gao, X., Liu, X., Wang, S., Liu, C., Yang, X., Li, Y. (2016). Filling a gap in the distribution of *Batrachochytrium dendrobatidis*: evidence in amphibians from northern China. *Diseases of Aquatic Organisms*, *118*(3), 259-265.
18. Coutinho, S.D., Burke, J.C., de Paula, C.D., Rodrigues, M.T., Catão-Dias, J.L. (2015). The use of singleplex and nested PCR to detect *Batrachochytrium dendrobatidis* in free-living frogs. *Brazilian Journal of Microbiology*, *46*(2), 551-555.
19. Wei, Y., Xu, K., Zhu, D.Z., Chen, X.F., Wang, X.L. (2010). Early-spring survey for *Batrachochytrium dendrobatidis* in wild *Rana dybowskii* in Heilongjiang province, China. *Diseases of Aquatic Organisms*, *92*(2-3), 241-244.
20. Conradie, W., Harvey, J., Kotzé, A., Dalton, D.L., Cunningham, M.J. (2011b). Confirmed amphibian chytrid in Mount Mulanje area, Malawi. *Herpetological Review*, *42*(3), 369-371.
21. Ocock, J.F., Rowley, J.J., Penma, T.D., Rayner, T.S., Kingsford, R.T. (2013). Amphibian chytrid prevalence in an amphibian community in arid Australia. *EcoHealth*, *10*(1), 77-81.
22. Ribeiro, J.W., Siqueira, T., DiRenzo, G.V., Lambertini, C., Lyra, M.L., Toledo, L.F., Haddad, C.F.B., Becker, C.G. (2020). Assessing amphibian disease risk across tropical streams while accounting for imperfect pathogen detection. *Oecologia*, *193*(1), 237-248.
23. Nava-González, B.A., Suazo-Ortuo, I., Parra-Olea, G., López-Toledo, L., Alvarado-Díaz, J. (2020). *Batrachochytrium dendrobatidis* infection in amphibians from a high elevation habitat in the trans-Mexican volcanic belt. *Aquatic Ecology*, *54*(1), 75-87.
24. Flechas, S.V., Sarmiento, C., Amézquita, A. (2012). *Bd* on the beach: high prevalence of *Batrachochytrium dendrobatidis* in the lowland forests of Gorgona Island (Colombia, South America). *EcoHealth*, *9*(3), 298-302.
25. Muletz Wolz, C.R., Yarwood, S.A., Campbell Grant, E.H., Fleischer, R.C., Lips, K.R. (2018). Effects of host species and environment on the skin microbiome of Plethodontid salamanders. *Journal of Animal Ecology*, *87*(2), 341-353.
26. Saenz, D., Hall, T.L., Kwiatkowski, M.A. (2015). Effects of urbanization on the occurrence of *Batrachochytrium dendrobatidis*: do urban environments provide refuge from the amphibian chytrid fungus? *Urban Ecosystems*, *18*(1), 333-340.
27. Savage, A.E., Grismer, L.L., Anuar, S., Onn, C.K., Grismer, J.L., Quah, E., Muin, M.A., Ahmad, N., Lenker, M., Zamudio, K.R. (2011). First record of *Batrachochytrium dendrobatidis* infecting four frog families from Peninsular Malaysia. *Ecohealth*, *8*(1), 121-128.
28. Petersen, C.E., Lovich, R.E., Phillips, C.A., Dreslik, M.J., Lannoo, M.J. (2016). Prevalence and seasonality of the amphibian chytrid fungus *Batrachochytrium dendrobatidis* along widely separated longitudes across the United States. *Ecohealth*, *13*(2), 368-382.
29. Schlaepfer, M.A., Sredl, M.J., Rosen, P.C., Ryan, M.J. (2007). High prevalence of *Batrachochytrium dendrobatidis* in wild populations of lowland leopard frogs *Rana yavapaiensis* in Arizona. *EcoHealth*, *4*(4), 421-427.
30. Ernetti, J.R., Boschetti, J.P., Delazeri, F., De Bastiani, V.I.M., Pontes, M.R., Ribeiro, L.P., Lingnau, R., Toledo, L.F., Lucas, E.M. (2020). High temporal and individual variation in the prevalence and intensity of chytrid infection in the southernmost Leaf Frog of the genus *Pithecopus* (Anura, Phyllomedusidae). *Hydrobiologia*, *847*(16), 3355–3364.
31. Van Sluys, M., Hero, J.M. (2010). How does chytrid infection vary among habitats? The case of *Litoria wilcoxii* (Anura, Hylidae) in SE Queensland, Australia. *EcoHealth*, *6*(4), 576-583.
32. Zumbado-Ulate, H., García-Rodríguez, A., Vredenburg, V.T., Searle, C. (2019). Infection with *Batrachochytrium dendrobatidis* is common in tropical lowland habitats: Implications for amphibian conservation. *Ecology and Evolution*, *9*(8), 4917–4930.
33. Rodríguez-Brenes, S., Rodriguez, D., Ibáñez, R., Ryan, M.J. (2016). Spread of amphibian chytrid fungus across lowland populations of Túngara frogs in Panamá. *PLoS One*, *11*(5), e0155745.
34. Voordouw, M.J., Adama, D., Houston, B., Govindarajulu, P., Robinson, J. (2010). Prevalence of the pathogenic chytrid fungus, *Batrachochytrium dendrobatidis*, in an endangered population of northern leopard frogs, *Rana pipiens*. *BMC Ecology*, *10*(1), 6.
35. Mendoza-Almeralla, C., López-Velázquez, A., Longo, A.V., Parra-Olea, G. (2016). Temperature treatments boost subclinical infections of *Batrachochytrium dendrobatidis* in a Mexican salamander (*Pseudoeurycea leprosa*). *Revista Mexicana de Biodiversidad*, *87*(1), 171-179.
36. Oficialdegui, F.J., Sánchez, M.I., Monsalve-Carcaño, C., Boyero, L., Bosch, J. (2019). The invasive red swamp crayfish (*Procambarus clarkii*) increases infection of the amphibian chytrid fungus (*Batrachochytrium dendrobatidis*). *Biological Invasions*, *21*(11), 3221-3231.
37. Kilburn, V.L., Ibáñez, R., Sanjur, O., Bermingham, E., Suraci, J.P., Green, D.M. (2010). Ubiquity of the pathogenic chytrid fungus, *Batrachochytrium dendrobatidis*, in anuran communities in Panamá. *EcoHealth*, *7*(4), 537-548.
38. Muletz, C., Caruso, N.M., Fleischer, R.C., McDiarmid, R.W., Lips, K.R. (2014). Unexpected rarity of the pathogen *Batrachochytrium dendrobatidis* in Appalachian *Plethodon* salamanders: 1957-2011. *PLoS One*, *9*(8), e103728.
39. Bai, C., Garner, T.W., Li, Y. (2010). First evidence of *Batrachochytrium dendrobatidis* in China: discovery of chytridiomycosis in introduced American bullfrogs and native amphibians in the Yunnan Province, China. *Ecohealth*, *7*(1), 127-134.
40. Goka, K., Yokoyama, J., Une, Y., Kuroki, T., Suzuki, K., Nakahara, M., Kobayashi, A., Inaba, S., Mizutani, T., Hyatt, A.D. (2009). Amphibian chytridiomycosis in Japan: distribution, haplotypes and possible route of entry into Japan. *Molecular Ecology*, *18*(23), 4757-4774.
41. Bataille, A., Fong, J.J., Cha, M., Wogan, G.O.U., Baek, H.J., Lee, H., Min, M.S., Waldman, B. (2013). Genetic evidence for a high diversity and wide distribution of endemic strains of the pathogenic chytrid fungus *Batrachochytrium dendrobatidis* in wild Asian amphibians. *Molecular Ecology*, *22*(16), 4196-4209.
42. Whitfield, S.M., Geerdes, E., Chacon, I., Ballestero Rodriguez, E., Jimenez, R.R., Donnelly, M.A., Kerby, J.L. (2013). Infection and co-infection by the amphibian chytrid fungus and ranavirus in wild Costa Rican frogs. *Diseases of Aquatic Organisms*, *104*(2), 173-178.
43. Picco, A.M., Collins, J.P. (2007). Fungal and viral pathogen occurrence in Costa Rican amphibians. *Journal of Herpetology*, *41*(4), 746-749.
44. Goldberg, C.S., Hawley, T.J., Waits, L.P. (2009). Local and regional patterns of amphibian chytrid prevalence on the Osa Peninsula, Costa Rica. *Herpetological Review*, *40*(3), 309.
45. Saenz, D., Adams, C. K., Pierce, J. B., Laurencio, D. (2009). Occurrence of *Batrachochytrium dendrobatidis* in an anuran community in the southeastern Talamanca region of Costa Rica. *Herpetological Review*, *40*(3), 311-313.
46. Sánchez, D., Chacón-Ortiz, A., León, F., Han, B. A., Lampo, M. (2008). Widespread occurrence of an emerging pathogen in amphibian communities of the Venezuelan Andes. *Biological Conservation*, *141*(11), 2898-2905.
47. Lannoo, M.J., Petersen, C., Lovich, R.E., Nanjappa, P., Phillips, C., Mitchell, J.C., Macallister, I. (2011). Do frogs get their kicks on Route 66? Continental US transect reveals spatial and temporal patterns of *Batrachochytrium dendrobatidis* infection. *PLoS One*, *6*(7), e22211.
48. Puschendorf, R., Hoskin, C.J., Cashins, S.D., McDonald, K., Skerratt, L.F., Vanderwal, J., Alford, R.A. (2011). Environmental refuge from disease-driven amphibian extinction. *Conservation Biology*, *25*(5), 956-964.
49. Kriger, K.M., Pereoglou, F., Hero, J.M. (2007). Latitudinal variation in the prevalence and intensity of chytrid (*Batrachochytrium dendrobatidis*) infection in eastern Australia. *Conservation Biology*, *21*(5), 1280-1290.
50. Stark, T., Laurijssens, C., Weterings, M., Martel, A., Köhler, G., Pasmans, F. (2017). Prevalence of *Batrachochytrium dendrobatidis* in a Nicaraguan, micro-endemic Neotropical salamander, *Bolitoglossa mombachoensis*. *Amphibia-Reptilia*, *38*(1), 102-107.
51. Lawson, T.D., Jones, M.L., Komar, O., Welch, A.M. (2011). Prevalence of *Batrachochytrium dendrobatidis* in *Agalychnis moreletii* (Hylidae) of El Salvador and association with larval jaw sheath depigmentation. *Journal of Wildlife Diseases*, *47*(3), 544-554.
52. Kriger, K.M., Hero, J.M. (2007). The chytrid fungus *Batrachochytrium dendrobatidis* is non-randomly distributed across amphibian breeding habitats. *Diversity and Distributions*, *13*(6), 781-788.
53. McMillan, K.M., Lesbarrères, D., Harrison, X.A., Garner, T.W. (2020). Spatiotemporal heterogeneity decouples infection parameters of amphibian chytridiomycosis. *Journal of Animal Ecology*, *89*(4), 1109-1121.
54. Sette, C.M., Vredenburg, V.T., Zink, A.G. (2020). Differences in fungal disease dynamics in co-occurring terrestrial and aquatic amphibians. *EcoHealth*, *17*(3), 302-314.
55. Kriger, K.M., Hero, J.M., Ashton, K.J. (2006). Cost efficiency in the detection of chytridiomycosis using PCR assay. *Diseases of Aquatic Organisms*, *71*(2), 149-154.
56. Peralta-García, A., Adams, A.J., Briggs, C.J., Galina-Tessaro, P., Valdez-Villavicencio, J.H., Hollingsworth, B.D., Shaffer, H.B., Fisher, R.N. (2018). Occurrence of *Batrachochytrium dendrobatidis* in anurans of the Mediterranean region of Baja California, México. *Diseases of Aquatic Organisms*, *127*(3), 193-200.
57. Hidalgo-Vila, J., Díaz-Paniagua, C., Marchand, M.A., Cunningham, A.A. (2012). *Batrachochytrium dendrobatidis* infection of amphibians in the Doñana National Park, Spain. *Diseases of Aquatic Organisms*, *98*(2), 113-119.
58. Ohst, T., Gräser, Y., Plötner, J. (2013). *Batrachochytrium dendrobatidis* in Germany: distribution, prevalences, and prediction of high risk areas. *Diseases of Aquatic Organisms*, *107*(1), 49-59.
59. Longo, A.V., Burrowes, P.A., Joglar, R.L. (2010). Seasonality of *Batrachochytrium dendrobatidis* infection in direct-developing frogs suggests a mechanism for persistence. *Diseases of Aquatic Organisms*, *92*(2-3), 253-260.
60. Forzán, M. J., Vanderstichel, R., Hogan, N. S., Teather, K., Wood, J. (2010). Prevalence of *Batrachochytrium dendrobatidis* in three species of wild frogs on Prince Edward Island, Canada. *Diseases of Aquatic Organisms*, *91*(2), 91-96.
61. Goldberg, T.L., Readel, A.M., Lee, M.H. (2007). Chytrid fungus in frogs from an equatorial African montane forest in western Uganda. *Journal of Wildlife Diseases*, *43*(3), 521-524.
62. Gründler, M.C., Toledo, L.F., Parra-Olea, G., Haddad, C.F., Giasson, L.O., Sawaya, R.J., Prado, C.P., Araujo, O.G., Zara, F.J., Centeno, F.C., Zamudio, K.R. (2012). Interaction between breeding habitat and elevation affects prevalence but not infection intensity of *Batrachochytrium dendrobatidis* in Brazilian anuran assemblages. *Diseases of Aquatic Organisms*, *97*(3), 173-184.
63. Terrell, V.C., Engbrecht, N.J., Pessier, A.P., Lannoo, M.J. (2014). Drought reduces chytrid fungus (*Batrachochytrium dendrobatidis*) infection intensity and mortality but not prevalence in adult crawfish frogs (*Lithobates areolatus*). *Journal of Wildlife Diseases*, *50*(1), 56-62.
64. Vredenburg, V.T., du Preez, L., Raharivololoniaina, L., Vieites, D.R., Vences, M., Weldon, C. (2012). A molecular survey across Madagascar does not yield positive records of the amphibian chytrid fungus *Batrachochytrium dendrobatidis*. *Herpetol Notes*, *5*, 507-517.
65. Weldon, C., du Preez, L., Vences, M. (2008). Lack of detection of the amphibian chytrid fungus (*Batrachochytrium dendrobatidis*) in Madagascar. *A Conservation Strategy for the Amphibians of Madagascar*. *Monografie XLV Torino*, *Museo Regionale di Scienze Naturali*, 95-106.
66. Pilliod, D.S., Muths, E., Scherer, R.D, Bartelt, P.E., Corn, P.S., Hossack, B.R., Lambert, B.A., McCaffery, R., Gaughan, C. (2010). Effects of amphibian chytrid fungus on individual survival probability in wild boreal toads. *Conservation Biology*, *24*(5), 1259-1267.
67. Rahman, M.M., Jahan, H., Rabbe, M.F., Chakraborty, M., Salauddin, M. (2021). First detection of *Batrachochytrium dendrobatidis* in wild frogs from Bangladesh. *Ecohealth*, *18*(1), 31-43.
68. Kolenda, K., Najbar, A., Ogielska, M., Balá, V. (2017). *Batrachochytrium dendrobatidis* is present in Poland and associated with reduced fitness in wild populations of *Pelophylax lessonae*. *Diseases of Aquatic Organisms*, *124*(3), 241-245.
69. Frías-Alvarez, P., Vredenburg, V.T., Familiar-López, M., Longcore, J.E., González-Bernal, E., Santos-Barrera, G., Zambrano, L., Parra-Olea, G. (2008). Chytridiomycosis survey in wild and captive Mexican amphibians. *Ecohealth*, *5*(1), 18-26.
70. Rebollar, E.A., Hughey, M.C., Harris, R.N., Domangue, R.J., Medina, D., Ibáñez, R., Belden, L.K. (2014). The lethal fungus *Batrachochytrium dendrobatidis* is present in lowland tropical forests of far eastern Panamá. *PLoS One*, *9*(4), e95484.
71. Richards-Hrdlicka, K.L., Richardson, J.L., Mohabir, L. (2013). First survey for the amphibian chytrid fungus *Batrachochytrium dendrobatidis* in Connecticut (USA) finds widespread prevalence. *Diseases of Aquatic Organisms*, *102*(3), 169-180.
72. Rowley, J.J., Chan, S.K., Tang, W.S., Speare, R., Skerratt, L.F., Alford, R.A., Cheung, K.S., Ho, C.Y., Campbell, R. (2007). Survey for the amphibian chytrid *Batrachochytrium dendrobatidis* in Hong Kong in native amphibians and in the international amphibian trade. *Diseases of Aquatic Organisms*, *78*(2), 87-95.
73. Mutnale, M.C., Anand, S., Eluvathingal, L.M., Roy, J.K., Reddy, G.S., Vasudevan, K. (2018). Enzootic frog pathogen *Batrachochytrium dendrobatidis* in Asian tropics reveals high ITS haplotype diversity and low prevalence. *Scientific Reports*, *8*(1), 1-11.
74. Bales, E.K., Hyman, O.J., Loudon, A.H., Harris, R.N., Lipps, G., Chapman, E., Roblee, K., Kleopfer, J.D., Terrell, K.A. (2015). Pathogenic chytrid fungus *Batrachochytrium dendrobatidis*, but not *B. salamandrivorans*, detected on eastern hellbenders. *PLoS One*, *10*(2), e0116405.
75. Jaeger, J.R., Waddle, A.W., Rivera, R., Harrison, D.T., Ellison, S., Forrest, M.J., Vredenburg, V.T., van Breukelen, F. (2017). *Batrachochytrium dendrobatidis* and the decline and survival of the relict leopard frog. *Ecohealth*, *14*(2), 285-295.
76. Rivera, B., Cook, K., Andrews, K., Atkinson, M. S., Savage, A. E. (2019). Pathogen dynamics in an invasive frog compared to native species.

*EcoHealth*, *16*(2), 222-234.

1. Labumbard, B.C., Shepack, A., Catenazzi, A. (2020). After the epizootic: Host–pathogen dynamics in montane tropical amphibian communities with high prevalence of chytridiomycosis. *Biotropica*, *52*(6), 1194-1205.
2. Zampiglia, M., Canestrelli, D., Chiocchio, A., Nascetti, G. (2013). Geographic distribution of the chytrid pathogen *Batrachochytrium dendrobatidis* among mountain amphibians along the Italian peninsula. *Diseases of Aquatic Organisms*, *107*(1), 61-68.
3. Hunter, D.A., Speare, R., Marantelli, G., Mendez, D., Pietsch, R., Osborne, W. (2010). Presence of the amphibian chytrid fungus *Batrachochytrium dendrobatidis* in threatened corroboree frog populations in the Australian Alps. *Diseases of Aquatic Organisms*, *92*(2-3), 209-216.
4. Erismis, U.C., Konuk, M., Yoldas, T., Agyar, P., Yumuk, D., Korcan, S.E. (2014). Survey of Turkey’s endemic amphibians for chytrid fungus *Batrachochytrium dendrobatidis*. *Diseases of Aquatic Organisms*, *111*(2), 153-157.
5. Hardman, R.H., Sutton, W.B., Irwin, K.J., McGinnity, D., Reinsch, S.D., Freake, M., Colclough, P., Miller, B.T., Da Silva Neto, J.G., Souza, M., Fitzpatrick, B., Miller, D.L. (2020). Geographic and individual determinants of important amphibian pathogens in hellbenders (*Cryptobranchus alleganiensis*) in Tennessee and Arkansas, USA. *Journal of Wildlife Diseases*, *56*(4), 803-814.
6. Keitzer, S.C., Goforth, R., Pessier, A.P., Johnson, A.J. (2011). Survey for the pathogenic chytrid fungus *Batrachochytrium dendrobatidis* in southwestern North Carolina salamander populations. *Journal of Wildlife Diseases*, *47*(2), 455-458.
7. Group, E.A., Pope, K.L., Wengert, G.M., Foley, J.E., Ashton, D.T., Botzler, R.G. (2016). Citizen scientists monitor a deadly fungus threatening amphibian communities in northern coastal California, USA. *Journal of Wildlife Diseases*, *52*(3), 516-523.
8. Valencia-Aguilar, A., Ruano-Fajardo, G., Lambertini, C., da Silva Leite, D., Toledo, L.F., Mott, T. (2015). Chytrid fungus acts as a generalist pathogen infecting species-rich amphibian families in Brazilian rainforests. *Diseases of Aquatic Organisms*, *114*(1), 61-67.
9. Aoust-Messier, A.M.D., Echaubard, P., Billy, V., Lesbarrères, D. (2015). Amphibian pathogens at northern latitudes: presence of chytrid fungus and ranavirus in northeastern Canada. *Diseases of Aquatic Organisms*, *113*(2), 149-155.
10. Canestrelli, D., Zampiglia, M., Nascetti, G. (2013). Widespread occurrence of *Batrachochytrium dendrobatidis* in contemporary and historical samples of the endangered *Bombina pachypus* along the Italian peninsula. *PloS One*, *8*(5), e63349.
11. Federici, S., Clemenzi, S., Favelli, M., Tessa, G., Andreone, F., Casiraghi, M., Crottini, A. (2008). Identification of the pathogen *Batrachochytrium dendrobatidis* in amphibian populations of a plain area in the Northwest of Italy. *Herpetology Notes*, *1*(1), 33-37.
12. Ficetola, G.F., Valentini, A., Miaud, C., Noferini, A., Mazzotti, S., Dejean, T. (2011). *Batrachochytrium dendrobatidis* in amphibians from the Po River Delta, Northern Italy. *Acta Herpetologica*,*6*(2), 297-302.
13. Kielgast, J., Rödder, D., Veith, M., Lötters, S. (2010). Widespread occurrence of the amphibian chytrid fungus in Kenya. *Animal Conservation*, *13*(1), 36-43.
14. Brannelly, L.A., Webb, R.J., Hunter, D.A., Clemann, N., Howard, K., Skerratt, L. F., Berger, L., Scheele, B.C. (2018). Non‐declining amphibians can be important reservoir hosts for amphibian chytrid fungus. *Animal Conservation*, *21*(2), 91-101.
15. Penner, J., Adum, G.B., McElroy, M.T., Doherty-Bone, T., Hirschfeld, M., Sandberger, L., Weldon, C., Cunningham, A.A., Ohst, T., Wombwell, E., Portik, D.M., Reid, D., Hillers, A., Ofori-Boateng, C., Oduro, W., Plötner, J., Ohler, A., Leaché ,A.D., Rödel, M.O. (2013). West Africa - a safe haven for frogs? A sub-continental assessment of the chytrid fungus (*Batrachochytrium dendrobatidis*). *PLoS One*, *8*(2), e56236.
16. Gower, D.J., Doherty-Bone, T.M., Aberra, R.K., Mengistu, A., Schwaller, S., Menegon, M., de Sá, R., Saber, S.A., Cunningham, A.A., Loader, S.P. (2012). High prevalence of the amphibian chytrid fungus (*Batrachochytrium dendrobatidis*) across multiple taxa and localities in the highlands of Ethiopia. *The Herpetological Journal*, *22*(4), 225-233.
17. Bower, D.S., Jennings, C.K., Webb, R.J., Amepou, Y., Schwarzkopf, L., Berger, L., Alford, R.A., Georges, A., Georges, D.T., Carr, L., Nason, D., Clulow, S. (2020). Disease surveillance of the amphibian chytrid fungus *Batrachochytrium dendrobatidis* in Papua New Guinea. *Conservation Science and Practice*, *2*(9), e256.
18. Lötters, S., Kielgast, J., Sztatecsny, M., Wagner, N., Schulte, U., Werner, P., Rödder, D., Dambach, J., Reissner, T., Hochkirch, A., Schmidt, B.R. (2012). Absence of infection with the amphibian chytrid fungus in the terrestrial Alpine salamander, *Salamandra atra*. *Salamandra*, *48*(1), 58-62.
19. Courtois, E.A., Pineau, K., Villette, B., Schmeller, D.S., Gaucher, P. (2012). Population estimates of *Dendrobates tinctorius* (Anura: Dendrobatidae) at three sites in French Guiana and first record of chytrid infection. *Phyllomedusa*: *Journal of Herpetology*, *11*(1), 63-70.
20. Doherty-Bone, T.M., Bielby, J., Gonwouo, N.L., LeBreton, M., Cunningham, A. A. (2008). In a vulnerable position? Preliminary survey work fails to detect the amphibian chytrid pathogen in the highlands of Cameroon, an amphibian hotspot. *The Herpetological Journal*, *18*(2), 115-118.
21. Alemu, J.B., Cazabon-Mannette, M.N., Cunningham, A.A., Dempewolf, L., Hailey, A., Mannette, R.P., Naranjit, K.T., Perkins, M.W., Schmidt-Roach, A.C. (2013). Presence of the chytrid fungus *Batrachochytrium dendrobatidis* in a vulnerable frog in Trinidad, west Indies. *Endangered Species Research*, *20*(2), 131-136.
22. Thien, T.N., Martel, A., Brutyn, M., Bogaerts, S., Sparreboom, M., Haesebrouck, F., Fisher, M.C., Beukema, W., Van, T.D., Chiers, K., Pasmans, F. (2013). A survey for *Batrachochytrium dendrobatidis* in endangered and highly susceptible Vietnamese salamanders (*Tylototriton* spp.). *Journal of Zoo and Wildlife Medicine*, *44*(3), 627-633.
23. Ouellet, M., Dejean, T., Galois, P. (2012). Occurrence of the amphibian chytrid fungus *Batrachochytrium dendrobatidis* in introduced and native species from two regions of France. *Amphibia-Reptilia*, *33*(3-4), 415-422.
24. Slough, B.G. (2009). Amphibian chytrid fungus in western toads (*Anaxyrus boreas*) in British Columbia and Yukon, Canada. *Herpetological Review*, *40*(3), 319.
25. Forrest, M.J., Edwards, M.S., Rivera, R., Sjöberg, J., Jaeger, J. (2015). High prevalence and seasonal persistence of amphibian chytrid fungus infections in the desert-dwelling Amargosa toad, *Anaxyrus nelsoni*. *Herpetological Conservation and Biology*, *10*(3), 917-925.
26. Harner, M.J., Merlino, J.N., Wright, G.D. (2013). Amphibian chytrid fungus in Woodhouse’s toads, plains leopard frogs, and American bullfrogs along the Platte River, Nebraska, USA. *Herpetological Review*, *44*(3), 459-461.
27. Gandola, R., Hendry, C.R. (2013). No detection of the chytrid fungus (*Batrachochytrium dendrobatidis*) in a multi-species survey of Ireland's native amphibians. *The Herpetological Journal*, *23*(4), 233-236.
28. Parrott, J.C., Shepack, A., Burkart, D., LaBumbard, B., Scime, P., Baruch, E., Catenazzi, A. (2017). Survey of pathogenic chytrid fungi (*Batrachochytrium dendrobatidis* and *B. salamandrivorans*) in salamanders from three mountain ranges in Europe and the Americas. *EcoHealth*, *14*(2), 296-302.
29. Ruggeri, J., Toledo, L.F., de Carvalho-e-Silva, S.P. (2018). Stream tadpoles present high prevalence but low infection loads of *Batrachochytrium dendrobatidis* (Chytridiomycota). *Hydrobiologia*, *806*(1), 303-311.
30. Pearl, C.A., Bowerman, J., Adams, M.J., Chelgren, N.D. (2009). Widespread occurrence of the chytrid fungus *Batrachochytrium dendrobatidis* on Oregon spotted frogs (*Rana pretiosa*). *EcoHealth*, *6*(2), 209-218.
31. Pullen, K.D., Best, A.M., Ware, J.L. (2010). Amphibian pathogen *Batrachochytrium dendrobatidis* prevalence is correlated with season and not urbanization in central Virginia. *Diseases of Aquatic Organisms*,*91*(1), 9-16.
32. Love, C.N., Winzeler, M.E., Beasley, R., Scott, D.E., Nunziata, S.O., Lance, S.L. (2016). Patterns of amphibian infection prevalence across wetlands on the Savannah River Site, South Carolina, USA. *Diseases of Aquatic Organisms*, *121*(1), 1-14.
33. Conradie, W., Weldon, C., Smith, K.G., Du Preez, L.H. (2011a). Seasonal pattern of chytridiomycosis in common river frog (*Amietia angolensis*) tadpoles in the South African Grassland Biome. *African Zoology*, *46*(1), 95-102.
34. Hossack, B.R., Adams, M.J., Grant, E.H.C., Pearl, C.A., Bettaso, J.B., Barichivich, W.J., Lowe, W.H., True, K., Ware, J.L., Corn, P.S. (2010). Low prevalence of chytrid fungus (*Batrachochytrium dendrobatidis*) in amphibians of US headwater streams. *Journal of Herpetology*, *44*(2), 253-260.
35. Greenbaum, E., Meece, J., Reed, K. D., Kusamba, C. (2015). Extensive occurrence of the amphibian chytrid fungus in the Albertine Rift, a Central African amphibian hotspot. *The Herpetological Journal*, *25*(2), 91-100.

**
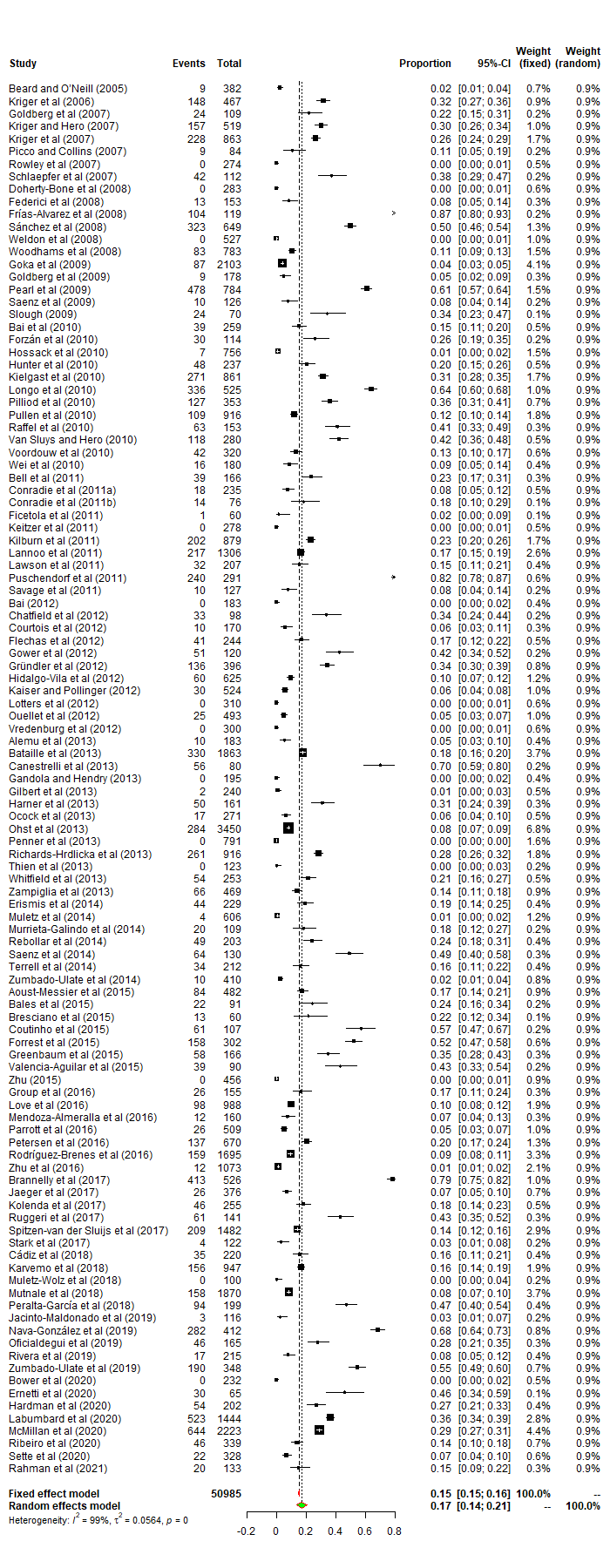
**

**Figure S1** Forest plot illustrating *Batrachochytrium dendrobatidis* infection in amphibians worldwide.

**
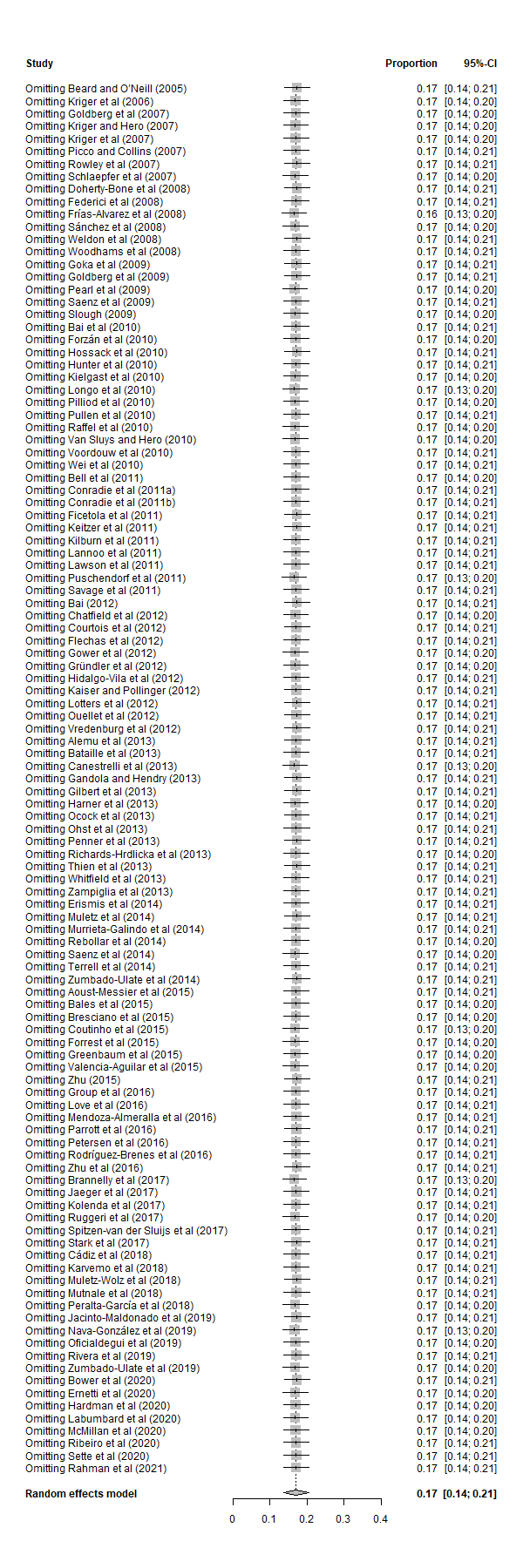
**

**Figure S2** Sensitivity analysis the stability of studies.
